# Identifying gene regulation modules associated with tumor metastasis using a network decomposition approach and combinatorial fusion analysis

**DOI:** 10.1371/journal.pone.0337873

**Published:** 2026-06-26

**Authors:** Aninda Astuti, Christina Schweikert, Chia-Wei Weng, Derbiau Frank Hsu, Ka-Lok Ng

**Affiliations:** 1 Department of Bioinformatics and Medical Engineering, Asia University, Taichung, Taiwan; 2 Division of Computer Science, Mathematics and Science, St. John’s University, New York, United States of America; 3 Department of Surgery, National Taiwan University Hospital and National Taiwan University College of Medicine, Taipei City, Taiwan; 4 Laboratory of Informatics and Data Mining, Department of Computer and Information Science, Fordham University, New York, United States of America; 5 AI and Quantum Research Center, Asia University, Taichung, Taiwan; 6 Department of Medical Research, China Medical University Hospital, China Medical University, Taichung, Taiwan; Public Library of Science, UNITED KINGDOM OF GREAT BRITAIN AND NORTHERN IRELAND

## Abstract

We systematically evaluated whether modular decomposition of molecular networks into gene regulatory modules (GRMs) enables the identification of metastasis‑associated genes. We developed an efficient bioinformatics framework that integrates subgraph extraction with combinatorial fusion analysis (CFA) to identify and prioritize metastasis‑associated GRMs in cancer networks. We validated top‑ranked GRMs using cancer hallmark annotations, enrichment analysis, drug–target associations, and survival data, and assessed GRM cooperativity through comparisons with prior metastasis studies. The proposed approach consistently outperformed existing methods in identifying metastasis‑associated GRMs. Robustness analyses across ten feature combinations and comparisons between three‑node and four‑node GRMs confirmed stable performance under diverse settings. Application to three independent KIRC metastasis cohorts further demonstrated improved identification of metastasis‑related GRMs. Overall, this integrated GRM‑based framework reliably captures coordinated regulatory patterns linked to metastasis and shows potential for identifying clinically relevant target genes and therapeutic drug candidates.

## Introduction

Gene cooperativity, whereby coordinated gene interactions produce effects not predictable from individual genes, is central to cancer progression and metastasis. Gene regulatory modules (GRMs) are widely used to model such coordinated activity within molecular networks. Previous studies have applied GRMs to cancer research using diverse strategies, including lung cancer gene networks [1], and tools like FDRnet, which employs Bayesian analysis to identify seed genes and form localized networks [2]. Huang et al. [3] developed a gene target network for lung cancer using the STRING database [4] to map interactions among differentially expressed genes and extract key modules. Gene synergy plays an essential role in tumor metastasis [5], with Xu et al. [6] showing that integrating gene regulation data into graph convolutional networks can effectively construct pan-cancer GRMs, using databases such as HumanNet v2 [7], KEGG [8], BioCarta [9] and Reactome [10] databases.

Interconnected gene modules are capable of performing specific cellular functions, with dynamic motifs like coupled feedback loops [11] enhancing both robustness and evolutionary potential. For instance, positive and negative feedback loops can induce oscillatory cell cycle behaviors [12], while studies on *E. coli* [13] have revealed how coupled feed-forward loops influence protein expression. These regulatory mechanisms are not only critical for normal cellular processes but also play a role in cancer progression.

Tumor metastasis, a primary cause of cancer-related deaths, is one such process where dysregulated gene networks contribute to malignancy. Identifying molecular biomarkers in these GRMs is crucial for advancing targeted therapies and improving diagnostics. Studies have uncovered various crosstalk between metastasis-associated pathways [14,15] and biomarkers, including microRNA markers in Kidney Renal Clear Cell Carcinoma (KIRC) [16] and gene markers in hepatocellular carcinoma (HCC) [17].

Current research employs multiple strategies to construct GRMs. First, use of protein-protein interactions (PPIs) and cluster analysis to identify metastasis-associated genes [18]. Second, the use of PPIs to infer GRMs and the application of graph theory to analyze network topology through metrics like degree, betweenness, and centrality. Centrality measures have been used to identify oncogenes [19], global regulators [20], glioblastoma biomarkers [21], and genes linked to drug resistance [22]. Third, *Weighted Gene Co-expression Network Analysis (WGCNA)* [23] was applied to detect clusters of highly correlated genes, yet it fails to reveal the regulatory interactions. For example, *WGCNA* has been utilized to identify diagnostic biomarkers for colorectal cancer [24] and markers associated with immune cell infiltration in kidney disease [25]. Fourth, single-cell data have been used in attempts to predict GRMs, these approaches continue to fall short in capturing regulatory relationships [26]. Fifth, multi-omics approach was proposed to construct GRMs for cancer cohorts but still lacking of regulatory information [27]. Sixth, studies are attempting to predict directed GRMs using single-cell multi-omics datasets by integrating scRNA transcriptomic and methylation data to predict GRMs for liver cancer and bladder based on back-propagation neural network [28]. The primary concern with this method is that several parameter choices lack clear justification—specifically, the formulation of the indicator equation, the selection of genes for constructing the final GRMs, and the measurement of gene importance via gene strength.

The above-mentioned approaches suggest that directional regulatory relationships between genes cannot be fully captured with high confidence [29]. Instead of relying on *WGCNA* or multi‑omics frameworks that assume such directionality, we adopt an *empirical* approach that decomposes networks into smaller, interpretable subgraphs (modules).

The concept of network motifs, introduced by Alon et al. [30], involves comparing a network to a randomized version of itself to identify sub-networks that occur more frequently than expected. However, this approach has limitations, including assumptions about motif frequencies and independence that may lead to false negatives [31]. To address this, we previously proposed a method that does not rely on the null model, using a network subgraph approach instead [32,33]. Originally referred to as motifs (renamed as subgraphs [32,33]), we now use the term GRMs to better capture their function.

We propose a network‑based framework that decomposes molecular interaction networks into GRMs and prioritizes their relevance to tumor metastasis. Building on our previous work [32], the approach leverages experimentally supported interactions to identify GRMs as potential metastasis‑associated and drug‑targetable modules.

Molecular networks are decomposed into four‑gene GRMs, with each gene annotated using four complementary ranking features. These features are integrated using combinatorial fusion analysis (CFA) [34–37] to prioritize metastasis‑associated GRMs. The modular design enables scalability by allowing smaller GRMs to be merged into larger regulatory units.

We validated the top‑ranked GRMs using cancer hallmark annotations, functional enrichment analyses, drug–target gene evaluation, and assessment of coordinated regulatory activity using the MET500 metastatic tumor cohort (https://met500.med.umich.edu/), demonstrating the framework’s ability to identify biologically meaningful, metastasis‑associated GRMs.

The next sections under ‘Methods’ will describe the workflow in detail. Then in the ‘Results and Discussions’, we will explore how to scale up from 4-node GRMs to larger modules. For this study, we selected renal cell carcinoma as the focus [38], identifying a set of 4-node GRMs within the KIRC network, each involving interactions between source and target nodes.

## Methods

**Fig 1:**
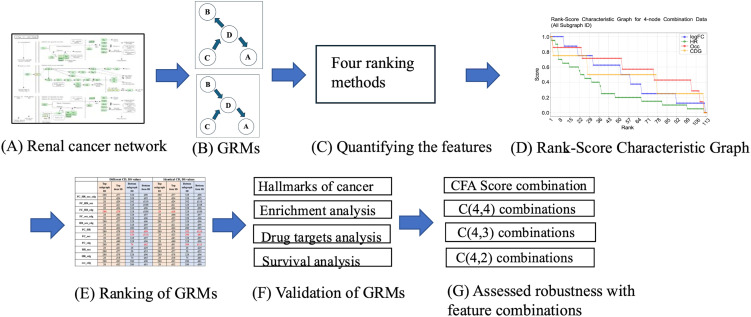
The workflow diagram for the study. **(A)** Renal cancer network (KIRC) retrieved from the KEGG database. **(B)** Utilized a subgraph method to extract GRMs. **(C)** Quantifying the four features. **(D)** The use of RSC graph for characterizing the four employed scoring methods. **(E)** Ranking of the GRMs. **(F)** Validation of GRMs. **(G)** Accessed robustness with feature combinations.

We present a network‑based framework to identify tumor metastasis–associated gene regulatory modules (GRMs). GRMs are extracted using a subgraph‑based approach and prioritized through combinatorial fusion analysis. Top‑ranked GRMs are systematically validated using cancer hallmark annotations, functional enrichment, drug–target relationships, and survival analysis. To assess added biological value, cooperative effects within GRMs are evaluated through comparisons with existing metastasis studies. An overview of the analytical workflow is shown in Fig 1.

### Data sources

Molecular network data were obtained from the KEGG database, which provides curated representations of biological systems spanning genomic, proteomic, metabolic, chemical, and drug‑related processes. Tumor metastasis cohorts were derived from the UCSC Xena platform and included both M0 and M1 samples, enabling computation of log₂ fold change (log(FC)) and hazard ratios (*HR*). In the KIRC cohort, there are 422 M0 samples and 79 M1 samples. M0/M1 is a clinical classification indicating the presence or absence of distant metastasis in the pathological staging system. The pathological stage distribution was as follows: stage I (n = 243), stage II (n = 54), stage III (n = 121), stage IV (n = 81), with two cases missing stage annotation.

To construct and characterize tumor metastasis–associated GRMs, we further evaluated identified GRMs using complementary tumor‑related ranking criteria. These included cancer driver gene (*cdg*) analysis and metastasis association evidence curated from three external databases— TMMGdb [39], HCMDB [40], and CMgene [41]—collectively referred to as *Occ*. Integration of molecular network structure with expression, survival, and metastasis annotations provided a comprehensive framework for prioritizing metastasis‑associated GRMs.

### Utilized a subgraph method to extract GRMs

In previous studies [32,33,42], we developed a subgraph approach, an alignment-free method, to analyze 71 molecular networks from KEGG. We decomposed each network into 3- and 4-node GRMs, including the KIRC network, to identify GRMs in the cancer network.

The process followed by the algorithm includes the following steps: (1) First, an adjacency matrix $A_{\text{net}}$ is constructed for the network to be analyzed, which consists of N nodes. The number of nodes incorporated into the subgraph (GRM) is set to four. (2) Four rows and four columns are extracted from $A_{\text{net}}$, generating C(N,4) possible combinations. These combinations form a new adjacency matrix, denoted as $\overset{\left(\text{4}\right)}{\underset{net}{A}}$. During this step, it is crucial that the row and column indices align appropriately to form $\overset{\left(\text{4}\right)}{\underset{net}{A}}$, and any disconnected subgraphs are removed. The total number of edges E within these subgraphs can vary, based on prior research study [43,44]. For example, a connected 4-node subgraph would have a minimum of three edges, i.e., E=3. (3) The matrix $\overset{\left(\text{4}\right)}{\underset{net}{A}}$ is then converted into integer values, and the number of edges $\overset{\left(\text{4}\right)}{\underset{net}{E}}$ present in this subgraph is counted. (4) This integer value typically represents an isomorphic form of a 4-node subgraph (GRM). The algorithm iteratively applies different permutation matrix operations until it identifies the smallest possible integer, which is then assigned a unique identifier labeled as $\overset{\left(\text{4}\right)}{\underset{net}{ID}}$. (5) To identify whether a matching subgraph exists based on its integer representation, subsets of integers corresponding to subgraphs with $\overset{\left(\text{4}\right)}{\underset{sub}{E}}$ edges (where $\overset{\left(\text{4}\right)}{\underset{sub}{E}}$ = $\overset{\left(\text{4}\right)}{\underset{net}{E}}$) must be selected. The integer identifier $\overset{\left(\text{4}\right)}{\underset{net}{ID}}$ is then compared with the corresponding identifier $\overset{\left(\text{4}\right)}{\underset{sub}{ID}}$ of any potential subgraph $\overset{\left(\text{4}\right)}{\underset{sub}{A}}$. A match between these integers confirms the detection of a 4-node subgraph (GRM) within the original matrix $A_{\text{net}}$. For a detailed explanation of the method, please refer to the prior research [42]. The KIRC network adjacency matrix is available in Supplementary File 9.

### Description of the four ranking methods

The first two ranking methods— log(FC) and *HR* score—were derived from our previous work [39]. TMMGdb uses DESeq2 [45] and Cox analysis to calculate log(FC) and *HR* by comparing RNAseq expression data between non-metastatic (M0) and metastatic (M1) samples. There is no universally accepted cutoff for |log(FC)|; thresholds can vary, with some studies using as low as |log(FC)| ≥ 0.20 [46] while others apply |log(FC)| ≥ 0.58 [47,48]. In this study, we adopt a threshold which is approximately equals to the average value; |log(FC)| = 0.38.

Differential expression was assessed using DESeq2, retaining genes with |log(FC)| ≥ 0.38 and adjusted p < 0.05. This threshold preserved 86% of the original dataset. The |log(FC)| range (0.38–0.86) was partitioned into ten equal intervals, with scores from 1 to 10 assigned accordingly and values ≥0.86 receiving the maximum score; higher scores indicate greater relevance to metastasis.

In the second method, GRM genes are ranked using *HRs* derived from Cox proportional hazards analysis. Genes with *HR* ≥ 1.05 and adjusted p < 0.05 are retained, while genes with *HR* < 1.05 are excluded and assigned a score of 0. The remaining *HR* values are partitioned into ten intervals and scored from 1 to 10, with higher scores indicating stronger associations with tumor metastasis.

Two complementary methods incorporate curated genetic evidence to evaluate metastasis relevance. First, we examined whether genes within each module appear in established tumor metastasis gene databases. Each gene was queried against three curated resources—TMMGdb (4,243 genes), HCMDB (1,938 genes), and CMgene (2,040 genes). Modules were assigned an occurrence (Occ) score ranging from 0 to 12, where a score of 12 indicates that all four genes are listed in all three databases.

Second, we assessed whether module components correspond to known cancer driver genes. This analysis evaluates the contribution of each four‑gene module to tumor metastasis, yielding a cancer driver gene (*cdg*) score ranging from 0 to 4.

For clarity, we use *FC, HR, Occ*, and *cdg* to represent log(*FC*), hazard ratio, the frequency of occurrence in the three metastasis gene databases, and cancer driver gene respectively.

### The CFA method in evaluating GRMs

After scoring the GRMs, the next step is to rank them based on the accumulated scores. This process is complicated by the need to combine continuous and categorical values from multiple criteria. To address this, we employ the combinatorial fusion analysis (CFA) framework, which facilitates the combination of different scoring methods. CFA simplifies the complexity arising from diverse scoring criteria and has been applied in various domains such as drug discovery [49], protein structure prediction [50], and the classification of documents according to the United Nations Sustainable Development Goals (SDGs) [51]. It has also been used to enhance data synthesis and prediction quality in the study of metal halide perovskites [52].

A key strength of CFA lies in its ability to integrate heterogeneous scoring functions without imposing model‑specific assumptions, making it well suited for aggregating multi‑criteria features in our analysis. In contrast, machine‑learning–based ranking methods require ground‑truth or relative ranking labels, which are unavailable in this setting. This distinction reflects a difference in problem formulation rather than a limitation of our approach: whereas ML ranking methods are inherently supervised, our study addresses an unsupervised ranking task. We therefore adopt CFA as an interpretable, label‑free framework and evaluate its reliability using perturbation‑based robustness analysis, which directly assesses ranking stability without relying on supervised validation.

Combining multiple scoring systems can be achieved through at least two approaches: score combination (*SC*) or rank combination (*RC*). Let *M* represent a set of *n* scoring methods, *M* = {*A*_*1*_*, …, A*_*i*_*,…,A*_*n*_} where *A*_*i*_ denotes the *i*-th scoring method. Consider a dataset *D* comprised of *m* items, *D* = {*d*_*1*_*,..., d*_*k*_*,...,d*_*m*_}. Each scoring method A∈M, considered here as a scoring system A, is described by a score function s_A_, rank function r_A_, and a rank-score characteristic (RSC) function f_A_. The score function for a system A, $s_{A_{i}}\left(d_{\alpha }\right),$ produces a real number or integer. Here, $s_{A_{i}}(d_{\alpha })$, represents the score assigned to item $d_{\alpha }$ by the *i-*th scoring method, A_i_.

Different scoring methods utilize varying metrics; therefore, it is crucial to establish standardized measures and use the normalized score functions for each method. The normalized score function $\overset{*}{\underset{A_{i}}{s}}\left(d_{\alpha }\right)\;$ for item $d_{\alpha }\;$ using the *i-th* scoring method is defined as:


$$\begin{array}{c} \overset{*}{\underset{A_{i}}{s}}(d_{\alpha })=(s\left(d_{\alpha }\right)-s_{min})/(s_{max}-s_{min})),\text{where i}=\text{1}\text{ to n and }\alpha =\text{1}\;to\;m \end{array}$$ (1)

where $s_{min}$ and $s_{max}$ represent the minimum and maximum scores, respectively. Normalizing the scores facilitates an effective comparison and integration of the different scoring methods, as they are each now within the range of 0–1.

The rank function for scoring system *A*, $r_{A\;}$, is generated by ordering the normalized score function $\overset{*}{\underset{A}{s}}\left(d_{\alpha }\right)$ in descending order and mapping the scores to ranks starting at 1 for the highest score. A rank-score characteristic (RSC) function for scoring system *A*, $f_{A\;}$, is a mapping from ranks to scores and is defined as:


$$\begin{array}{c} f_{A_{i}}\left(d_{\alpha }\right)=\overset{*}{\underset{A_{i}}{s}}\left(\overset{-\text{1}}{\underset{A_{i}}{r}}\left(d_{\alpha }\right)\right),\text{where i}=\text{1}\text{ to n and }\alpha =\text{1}\;to\;m\; \end{array}$$ (2)

After selecting *n* scoring methods, the average score combination method for data d_α_ is given by:


$$\begin{array}{c} S_{ASC}\left(d_{\alpha }\right)=\;\frac{\sum_{A\in S}\overset{*}{\underset{A}{s}}\left(d_{\alpha }\right)}{\left|S\right|},\text{ where S }\subseteq \text{ M and }\alpha =\text{1}\;to\;m \end{array}$$ (3)

$S_{ASC}\left(d_{\alpha }\right)$ indicates that the score for data $d_{\alpha }$ is the average of the scores from *n* scoring methods.

The aforementioned method involves averaging the scores from four different scoring methods, each with equal weight. However, this approach may introduce bias, as it doesn’t account for the varying degrees of differences among the scoring systems. One solution is to employ cognitive diversity (*CD*) [53,54] to compute a metric that can be used as a weight for the scoring systems. The *CD* metric uses the area between the rank-score characteristic (RSC) functions of two scoring systems to represent the diversity. The *CD* metric quantifies the difference in scoring behavior between two scoring systems, which is distinct from the Pearson correlation in classical statistics which compares two sets of scores.

The cognitive diversity (*CD*) between two scoring methods, *A* and *B* is computed using the formula in Equation 4.


$$\begin{array}{c} CD(A,B)=CD\left(f_{A,\;}f_{B\;}\right)=\;\sqrt{\sum_{i=\text{1}}^{m}((f_{A}(i)-{f_{B}(i))}^{\text{2}}/m},\;where\;A,B\in M \end{array}$$ (4)

The greater the *CD (diversity)* between two scoring systems, the larger the difference in their scoring behavior. Subsequently, we calculate the diversity strength (*DS*) metric, which represents the average *CD* between the scoring method *A* and other scoring methods, calculated using the following formula:


$$\begin{array}{c} DS(A)=\frac{\sum_{\begin{array}{c} @lB\in M\\ B\neq A \end{array}}\;\;\;CD(A,B)}{|M|-\text{1}},\text{ where A }\in \text{ M} \end{array}$$ (5)

We observed that GRMs frequently share the same ranking for the cancer driver gene (*cdg*) score due to the score’s range of 0–4, which often results in identical ‘*cdg*’ scores for different GRMs. Consequently, we have decided to focus on the weighted score combination method. The weighted score combination $S_{WSC}\left(d_{\alpha }\right)$ can be obtained from the score function $\overset{*}{\underset{A_{i}}{s}}$ using *DS* as the weight, which is defined by Equation 6 below


$$\begin{array}{c} S_{WSC}\left(d_{\alpha }\right)=\;\frac{\sum_{A\in S}DS(A)\overset{*}{\underset{A}{s}}(d_{\alpha })}{\sum_{A\in S}\;DS(A)}\;,\text{ where S }\subseteq \text{ M and }\alpha =\text{1}\;to\;m\; \end{array}$$ (6)

For this study, we will employ the aforementioned methods to rank the GRMs. In summary, combinatorial fusion analysis (CFA) is a framework that uses the Rank-Score Characteristic (RSC) function to measure *CD* between two scoring systems and provides methods for scoring system combinations. *CD* and *DS* measure the dissimilarity or inconsistency between scoring methods.

### Applying multiple score combinations to rank the GRMs

An important step in the analysis is combining the four existing scoring methods. This approach has been explored in previous studies to improve multivariate or multi-objective classification, prediction, and optimization [54–56]. There are 11 possible combinations of the four ranking methods, each yielding a weighted score combination (*SC*) by *DS*. Using KIRC as an example, we construct a 114 x 11 matrix where each row corresponds to a GRM, and each column represents the rankings derived from one of the 11 combinations. A second matrix is created where rows indicate rankings from 1 to 114, and columns show rankings from the 11 models. Modules that rank within the top five across these combinations are selected for further analysis.

### Assess the relevance of the GRMs related to KIRC using GSEA and GOEA

To validate the significance of the ranked 4-node GRMs, we retrieve direct protein-protein interaction (PPI) partners for each genetic element from the STRING database. Enrichment analysis is then performed using DAVID, focusing on the Wikipathways and DisGeNET annotations. We examine 4-node GRMs using rankings from both average and weighted score combinations, excluding rank combinations where some methods only allow a limited number of ranks, such as the cancer driver gene ranking.

The Molecular Signatures Database (MSigDB) [57] organizes gene sets into collections, including the cancer-related hallmark gene set. For this study, we used the hallmark gene set from MSigDB (v2024.1.Hs, August 2024), which includes 50 gene sets. Gene Set Enrichment Analysis (GSEA) is employed to analyze the significance of the GRMs in relation to these hallmark pathways.

The Wikipathways annotation in DAVID provides access to biological pathways curated by the scientific community, helping to visualize and analyze gene functions and interactions within these pathways. Additionally, the DisGeNET database [58] supports the identification of gene-disease associations, offering valuable insights into the genetic basis of human diseases from a variety of sources, including expert-curated repositories and GWAS catalogs.

### Assess the relevance of the GRMs related to KIRC using drug-target information and survival analysis

Determining whether GRMs are involved in critical signaling pathways is essential, as their dysregulation can lead to uncontrolled cell growth and cancer formation. The KEGG drug database (https://www.genome.jp/kegg/drug/) is used to assess whether the GRMs are associated with known drug compounds. This resource includes information on approved drugs in Japan, the USA, and Europe, and links drugs to their therapeutic targets, molecular interactions, and drug metabolism, which can serve as potential biomarkers for diagnosis and prognosis.

The survival analysis results for the top and bottom ranked GRMs are shown in the Kaplan-Meier (KM) plots, which are derived from queries in the UALCAN database [59,60].

## Results and discussions

### Robustness of GRM ranking and comparison with single‑gene baselines

To support the choice of |log(FC)| = 0.38, we evaluated the robustness of GRM rankings under symmetric negative and positive perturbations of this threshold. Perturbations were applied in 5% increments from −15% to +15%, with additional extreme cases at −31.6% and +31.6%. The results reveal ranking‑dependent robustness: the top‑5 GRMs remain highly stable across all perturbations, including extreme shifts, whereas the top‑10 GRMs show progressively reduced stability as perturbation magnitude increases. These findings justify the selected threshold and support the robustness of the proposed ranking framework.

To assess whether GRM‑based findings could be reproduced by single‑gene ranking, we performed a baseline analysis using individual log₂ logFC and HR metrics. Genes were filtered according to two criteria: (i) |logFC| ≥ 0.38 with adjusted p < 0.05, and (ii) HR ≥ 1.05 with adjusted p < 0.05. From criterion (i), the top‑10 upregulated and bottom‑10 downregulated genes were selected, whereas from criterion (ii), the top‑10 genes with the largest HR values were retained. None of these 30 genes overlapped with those comprising the top two ranked GRMs (Table 2), indicating that the GRM framework captures coordinated regulatory patterns not identifiable through single‑gene ranking alone.

**Table 2:** The subgraph IDs and gene symbols of the top two (highest and second highest) and bottom two (lowest and second lowest) ranked 4-node GRMs (both weighted and average *DS* are used for *SC* ranking). The topology of the top two and bottom two ranked 4-node GRMs, namely ID_28, ID_280, and ID_328, is presented in Supplementary File 2.

	ID	Item	Frequency	Official gene symbol
Highest ranked	280	*d77* *d19* ^*ave*^	7	*TGFB1|EGLN2|ARNT|EPAS1* *TGFB1|PDGFB|ARNT|EPAS1* ^*ave*^
Second highest ranked	28	*d24* *d25* ^*ave*^	6	*TGFB1|TGFA|EGLN2|EPAS1* *TGFB1|TGFA|ARNT|EPAS1* ^*ave*^
Lowest ranked	328	*d96*	6	*RAP1A|CRK|GAB1|RAPGEF1*
Second lowest ranked	328	*d95*	6	*RAPGEF1|GAB1|MET|CRK*

### The Rank-Score Characteristic (RSC) Graph, cognitive diversity and diversity strength

Upon decomposing the KIRC network into 4-node GRMs, a limited variety of GRM types were identified; that is, GRMs with large graph energies are not present [32,33], rather than the full spectrum of 199 possibilities. The specific GRMs found include types: {d_1_, d_2_, …, d_11_} = {14, 28, 74, 76, 92, 280, 328, 344, 392, 394, 2184}, as detailed in Supplementary File 1. The complete topology of all potential 4-node GRMs is available in Supplementary File 2.

Should any genetic element within a GRM be without a feature score value, the GRM is excluded, leaving 114 for analysis. Our previous research [32] noted the absence of certain GRM patterns, with the missing types linked to significant graph energies. Networks of molecular interactions are built from a finite number of regulatory topologies.

**Fig 2:**
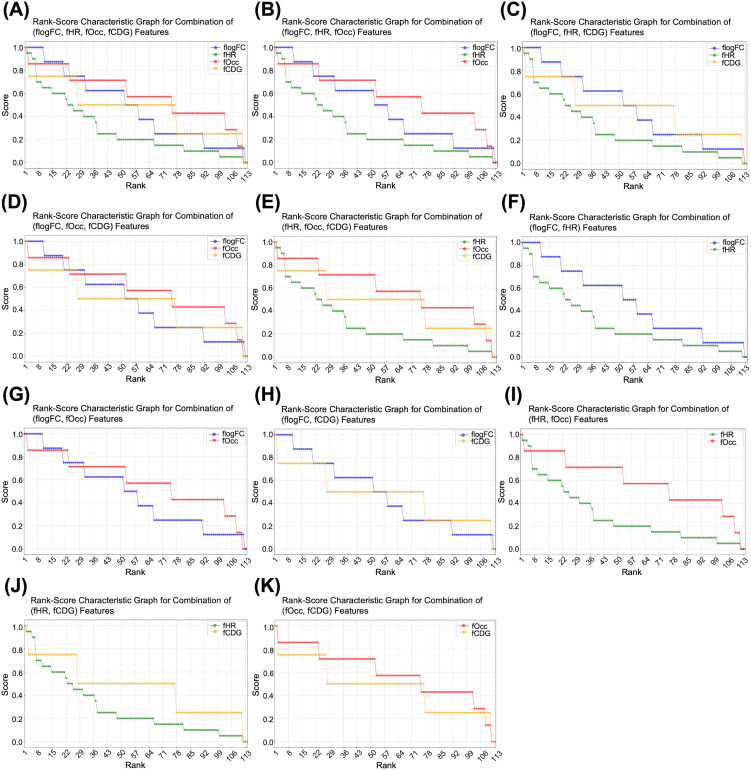
The Rank-Score Characteristic Graph for the 11 combinations of scoring methods. **(A)** The four features *log(FC), HR*, *Occ* and *cdg*. (B) *log(FC), HR* and *Occ*. (C) *log(FC), HR* and *cdg*. (D) *log(FC)*, *Occ* and *cdg.* **(E)** *HR*, *Occ* and *cdg.* (F) *log(FC)* and *HR*. (G) *log(FC)* and *Occ*. (H) *log(FC)* and *cdg.* **(I)** *HR* and *Occ*. **(J)** *HR* and *cdg.* **(K)** *Occ* and *cdg.*

We opted for a thorough examination of the 4-node GRMs over the 3-node counterparts due to the nearly doubled quantity, offering more detailed insights. Fig 2 presents the RSC graph for the four employed scoring methods.

Table 1 outlines the *CD* scores derived from the six possible combinations of two out of the four scoring systems. The greatest distance observed is 0.329 between the *HR* and *Occ* plots, suggesting these methods assess GRMs differently. In contrast, the smallest distances are both 0.144, found between the *cdg* and *FC* plots and the *cdg* and *Occ* plots, indicating similar assessments by these methods.

**Table 1:** The *CD* values for the six possible pairs of models, where *CD(X,Y)* represents the diversity between ranking methods *X* and *Y*, where *X* and *Y* are elements of {*FC* (equivalent to *log(FC)), HR, Occ*, *cdg*}.

*CD(X,Y)*	*CD(FC,HR)*	*CD(FC,Occ)*	*CD(FC,cdg)*	*CD(HR,Occ)*	*CD(HR,cdg)*	*CD(Occ,cdg)*
*CD* value	0.223	0.172	0.144	0.329	0.219	0.144

We then computed the *DS* metric to represent the average *CD* across different scoring methods. Supplementary information (Supplementary Table 1) lists the *DS* values for 11 possible scoring methods, with *DS_Z* denoting the DS between scoring method Z and the other scoring methods.

The *HR* feature’s *DS* value is the largest at 0.257, suggesting its distinct rating approach. This is supported by the *DS_HR* column in Supplementary information (Supplementary Table 1), which shows the largest distances. When comparing only two scoring methods, the *DS* value is symmetric: *DS(X, Y) = DS(Y, X).*

Table 2 compares weighted score combination (*SC*) by *DS* and average score combination in GRM ranking, focusing the top two and bottom two ranked GRMs, the rationale being to uncover GRMs exhibit different biological functions.

When using four features (*FC_HR_Occ_cdg*), both weighted *SC* and average *SC* are applied. For the other 10 combinations, we recalculated the weighted score values for each combination using the same *DS* values derived from the *FC_HR_Occ_cdg* combination for ranking (Supplementary information – Supplementary Table 2). The results of *the highest and lowest five ranked GRMs for the 11 combinations* is summarized in Supplementary information (Supplementary Table 3).

An alternative method for selecting the top and bottom ranked GRMs is to identify GRMs that most frequently appear as the highest-ranked and second highest-ranked across the 11 combinations. The results show that item *d24* appears most frequently as the highest-ranked (frequency = 3), and item *d21* as the second-highest-ranked (frequency = 2). Notably, the above-mentioned items differ from the items *d77* and *d18* listed in Table 2. Among the lowest-ranked items across 11 combinations, *d95* appears three times, while *d99* and *d108* each appear twice. Notably, *d95* consistently ranks lowest in this context.

### To assess the relevance of the GRMs related to renal cancer using Hallmarks of cancer

Supplementary information (Supplementary Table 4) summarizes the top three significant cancer hallmark annotations (p-value < 0.05) for the top two and bottom two 4-node GRMs. While the p-values for these cancer hallmark annotations exceed 0.05 in some cases, a clear distinction between the top two and bottom two GRMs emerges.

The top two GRMs show a direct association with cancer pathways, while only the second-lowest ranked GRM displays a significant association with cancer hallmark formation. The lowest-ranked GRM, however, lacks any significant hallmark annotation, further reinforcing that our method is capable of distinguishing GRMs related to tumor metastasis from other network components within the KIRC network.

When applying an alternative method to select the top (*d24, d21*) and bottom (*d95, d99, d108*) ranked GRMs, the hallmark annotations also highlight a notable difference between the highest two ranked and lowest two ranked GRMs. Supplementary information (Supplementary Tables 4 and 5) present nearly identical findings, with the addition of “IL6 JAK STAT3 SIGNALING” and “COAGULATION” found in Supplementary information (Supplementary Table 4). This consistency between both scoring methods confirm the robustness and reliability of the results.

### Further assessment of the GRMs’ relevance to KIRC using GOEA

Supplementary information (Supplementary Table 6) provides a summary of the highest two and lowest two ranked 4-node GRMs, including their enriched pathways (Wikipathways annotations) and associated diseases (DisGeNET annotations). These annotations reflect a significant biological difference between the top two and bottom two ranked GRMs. Specifically, the top two GRMs are closely related to renal cell carcinoma and cancer pathways, while the bottom two modules predominantly show annotations that are unrelated to cancer formation, with only the second annotation being associated ‘MET in type 1 papillary renal cell carcinoma’. Supplementary Files 3 and 4 provides a comprehensive list of the results of GOEA for the top two and bottom two ranked GRMs.

Among the DisGeNET annotations, the highest-ranked GRM reveals a connection to hereditary paraganglioma-pheochromocytoma (PGL/PCC) syndromes, which involve tumors that grow along the spine or adrenal glands. PGL/PCC is characterized by paragangliomas, often found at the carotid artery split in the upper neck. In contrast, the second-highest ranked GRM is linked to glioblastoma multiforme and hereditary PGL/PCC syndrome, highlighting a strong association with tumorigenesis.

The bottom two ranked GRMs, however, exhibit weaker tumor-related annotations. While the lowest ranked GRM shows no connection to tumors, the second-lowest GRM reveals associations with neoplasm invasiveness (the ability of tumors to invade surrounding tissue) and squamous cell carcinoma (skin cancer).

This suggests that the top-ranked GRMs are significantly more associated with tumor formation than the bottom-ranked GRMs, further validating the effectiveness of our method in isolating tumor metastasis-associated GRMs.

We also calculated *JI* to compare the top and bottom ranked GRMs for the following two scenarios (i) we re-calculate the *CD* and *DS,* (ii) we use the original *CD* and *DS*. This was done to investigate whether the ranking of GRMs is robust across variations in the analysis method. The *JI* values for the top five and bottom five ranked GRMs were computed for both “different *CD, DS*” and “same *CD, DS*” scenarios. Interestingly, the *JI* values indicated minimal change in the GRM rankings when moving from five to ten items, suggesting that the ranking method is stable and consistent.

*JI* calculation for the top 5, bottom 5 data items for (i) different *CD*, *DS,* (ii) same *CD, DS*. Repeat that *JI* for top 10 and bottom 10. Then we can see if the *JI* changes a lot from 5 to 10 items, if there is not much change this suggests that the use of (i) and (ii) does not affect the ranking or its robustness.

To further validate the CFA method, we considered GRMs composed of three genetic elements (3-node GRMs). The results of the GOEA also showed a disparity between the top-ranked GRMs and the bottom-ranked GRMs (Supplementary information – Supplementary Table 7, Supplementary File 6). Again, the top two GRMs are closely related to renal cell carcinoma and cancer pathways, while the bottom two GRMs predominantly show annotations that are unrelated to cancer formation, with only the lowest ranked GRM associated ‘MET in type 1 papillary renal cell carcinoma’. This reinforces the validity of the CFA method, as the results align with those derived from 4-node GRMs.

### Relevance of GRMs to drug targets of KIRC cohort

In Table 3, we list drugs associated with the treatment of renal cancer or anemia linked to chronic renal disease. A striking finding is that four out of five genes (80%) targeted for renal cancer treatment are linked to the top two ranked GRMs, reinforcing the clinical relevance of our identified modules. Furthermore, we found no drug associations for the bottom two ranked GRMs, further indicating that the top GRMs have stronger potential for therapeutic applications.

**Table 3:** The drugs (USAN/INN/TN) recorded by the KEGG drug database that are used for the treatment of renal cancer or anemia associated with chronic renal disease.

Highest ranked GRM				Second highest ranked GRM			
*Gene symbol: TGFB1|EGLN2|ARNT|EPAS1* *KEGG name: TGF-β|HPH|HIF-β |HIF-α*				*Gene symbol: TGFB1|PDGFB|EGLN2|EPAS1* *KEGG name: TGF-β|TGF-a|HPH|HIF-α*			
*TGFB1*	*EGLN2*	*ARNT*	*EPAS1*	*TGFB1*	*TGFA*	*EGLN2*	*EPAS1*
*Fresolimumab Livmoniplimab Linavonkibart*	*Roxadustat, Daprodustat, Vadadustat, Molidustat sodium, Enarodustat, Molidustat*	*NA*	*Belzutifan*	*Fresolimumab Livmoniplimab Linavonkibart*	*Fepixnebart*	*Roxadustat, Daprodustat, Vadadustat, Molidustat sodium, Enarodustat*	*Belzutifan*

### Survival analysis of KIRC cohort using the UALCAN database

**Fig 3:**
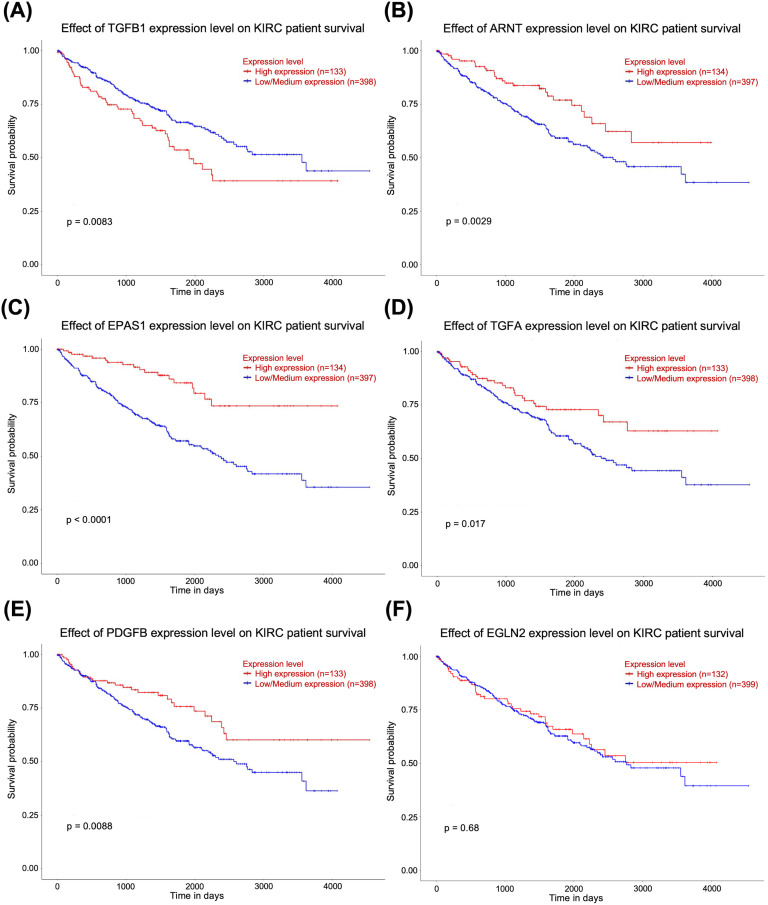
The results of the survival probability analyses, (A) *TGFB1* (B) *ARNT,* (C) *EPAS1* (D) *TGFA,* *(E)* *PDGFB* and (F) *EGLN2.* There is statistically significant negative impact on survival probability, leading to lower survival rates.

We conducted survival analyses to study the gene cooperativity of the identified GRMs and compared them with existing findings. The first analysis was performed using the UALCAN database, and the second utilized the cBioPortal database [61]. The results of the survival analysis using the UALCAN database [59,60] are shown in Fig 3, highlighted that high expression of *TGFB1* and low expression of *ARNT, EPAS1, TGFA*, and *PDGFB* are significantly associated with lower survival rates in KIRC patients. Specifically, five out of six genes (83.3%) identified as risk biomarkers show a negative impact on survival, while *EGLN2* (Fig 3 (F)) did not show any significant survival impact. Conversely, among the eight genes in the five lowest-ranked GRMs, only one gene, *SOS1*, impacted survival probability (Supplementary information – Supplementary Figure 1). These findings further emphasize that the top-ranked GRMs are more closely associated with survival outcomes and tumor metastasis.

### Survival analysis of KIRC cohort using the cBioPortal databases and metastasis-associated genes (*Occ*)

The results of the survival analysis using the highest two and lowest two GRMs, conducted through the cBioPortal database, are shown in Table 4. We tested the consistency of the results by repeating the analysis three times with different TCGA datasets: (i) TCGA Firehose Legacy, (ii) TCGA Nature 2013, and (iii) TCGA GDC.

**Table 4:** Survival analysis was conducted using the cBioPortal database for the two highest- and two lowest-ranked GRMs, as well as four additional genes from reference [18]. The symbols ‘Y’ and ‘NS’ indicate p-values of <0.05 and >0.05, respectively. Genes related to metastasis are denoted by the abbreviation ‘*Occ’.*

Genes	Official gene symbol	Firehose	Nature2013	GDC	*Occ*
Highest ranked	*TGFB1, PDGFB, ARNT, EPAS1* ^*ave*^	Y	Y	Y	11
Second highest ranked	*TGFB1, TGFA, ARNT, EPAS1* ^*ave*^	Y	Y	Y	11
Lowest ranked	*RAP1A, CRK, GAB1, RAPGEF1*	Y	Y	NS	9
Second lowest ranked	*RAPGEF1, GAB1, MET, CRK*	Y	Y	NS	8
metastasis- associated genes [18]	*COL1A1, DCN, FBLN1, POSTN*	NS	NS	NS	7
	*COL1A2, DCN, FBLN1, POSTN*	NS	NS	NS	6
	*COL5A1, DCN, FBLN1, POSTN*	Y	NS	NS	4
	*COL6A3, DCN, FBLN1, POSTN*	Y	NS	NS	4
	*COL1A1, COL5A1, COL6A3, DCN*	Y	NS	Y	4
	*COL1A1,COL5A1,COL6A3,FBLN1*	NS	Y	NS	4
	*COL1A1,COL5A1,COL6A3,POSTN*	Y	NS	Y	5
	*COL1A1,COL1A2,COL5A1,COL6A3*	Y	NS	Y	5
metastasis- associated genes [62]	*VHL, PBRM1, BAP1, SETD2*	NS	NS	NS	7
metastasis- associated genes [63]	*ABCG1, HAVCR2, CD14, TGFA*	NS	S	NS	7

The Genomic Data Commons (GDC) utilizes harmonized clinical data, which may alter survival times, censoring status, or event definitions. The other two datasets might have outdated or less complete clinical follow-up, which can inflate or deflate significance.

We emphasize that our survival analysis was performed using a combination of four biomarkers, rather than a single one. This analysis fundamentally differs from methods that rely on four features—*logFC*, *HR, Occ* and *cdg*—which are attributes of individual biomarkers. Thus, our study highlights the cooperative effect of a set of biomarkers, which should not be equated with the effects of individual biomarkers.

Additionally, we compared our findings with 3 independent KIRC cohort studies, which identified seven genes [18]—namely, the collagen family (*COL1A1, COL1A2, COL5A1*, and *COL6A3*), *DCN, FBLN1, and POSTN*—that were significantly upregulated in metastatic tumors compared to primary tumors. Another study [62] reported four genes—*VHL*, *PBRM1*, *BAP1*, *SETD2*—as key drivers of tumor initiation and metastasis. The third study by [63] identified increased mRNA expression of four genes—*ABCG1, HAVCR2, CD14, TGFA*—in KIRC samples for prognostic genes, relative to adjacent control tissue.

Table 4 summarizes the survival analysis results using the cBioPortal database for the top two and bottom two ranked GRMs (derived from average *SC*, as shown in Table 2), along with four genes selected from the seven genes [18], which include one gene from the collagen family and *DCN, FBLN1,* and *POSTN*.

Different combinations of the seven genes were tested to identify which sets of four genes yield consistent survival analysis results. First, one gene from the collagen family was selected and combined with the three non-collagen genes (*DCN, FBLN1,* and *POSTN*). Second, a different collagen gene was chosen and again combined with the same three non-collagen genes. Third, three collagen genes (*COL1A1, COL5A1, COL6A3*) were each combined with one of the non-collagen genes to form three additional gene sets.

Table 4 presents the survival analysis results. The top two ranked GRMs, based on average SC, consistently produced statistically significant p-values (<0.05) across all three TCGA datasets. In contrast, the bottom two ranked GRMs had at least one non-significant (NS) result. Gene sets selected from Reference [49] also showed mostly NS results, with at least two NS outcomes observed in four out of eight cases. It was also found that the key drivers of tumor initiation and metastasis yield NS results in survival analyses across all three TCGA datasets.

Furthermore, genes related to metastasis, ‘*Occ’*, are relative fewer compared to the highest two ranked GRMs. We noted that the lowest two ranked GRMs have a moderate number of ‘*Occ’*, this could explain why the survival analysis difference between the highest two and lowest two are moderate.

We compared our findings with three independent KIRC cohort studies. Survival analysis, conducted using a combination of four biomarkers, indicates that our current approach is more effective in identifying GRMs associated with metastasis.

### Quantifying the difference for the weighted *SC* and average *SC* results using Jaccard index

To quantify the differences in GRM rankings derived from using weighted *SC* and average *SC* methods, we calculated the *JI* for various subsets of the ranked GRMs. For the top five, bottom five, top ten and bottom ten ranked GRMs, we observed that both methods identified a similar set of GRMs, with *JI* values of 2/3 for all these subsets, except for a bottom‑10 cases where the *JI* = 0.538. This suggests that the weighted *SC* and average *SC* methods produce nearly identical results for these GRM rankings.

The *JI* analysis for additional combinations (provided in Supplementary File 5) showed that for most cases, the *JI* was exactly one for the top five and bottom five ranked GRMs, indicating complete agreement between the weighted *SC* and average *SC* methods. This further underscores the reliability of the results obtained using both methods.

### To assess the relevance of the 3-node GRMs related to renal cancer

In addition to the 4-node GRMs, we also examined 3-node GRMs (Supplementary information – Supplementary Table 7) to assess whether the top and bottom ranked GRMs demonstrated distinct biological functions. The comparison between the 3-node and 4-node GRMs (Supplementary information – Supplementary Table 7 and Table 2) revealed that the 4-node GRMs encompass all the 3-node GRMs, except for the second-lowest ranked 4-node GRM, where the gene *RAP1A* is absent. This suggests a high level of consistency between the results of the 4-node and 3-node GRMs, further supporting the validity of our method in identifying metastasis-associated GRMs.

### Module-Level Co-expression Analysis in the MET500 Cohort

To assess coordinated regulatory activity in metastatic tumors, we analyzed gene expression data obtained from the MET500 cohort (https://met500.med.umich.edu/) for the top- and bottom-ranked GRMs. The MET500 cohort is a pan‑cancer dataset focused on advanced and metastatic disease. Unlike primary tumor resources, it captures real‑world metastatic biopsies and integrates clinical, pathological, and genomic data to enable comprehensive characterization of tumor metastasis.

**Fig 4:**
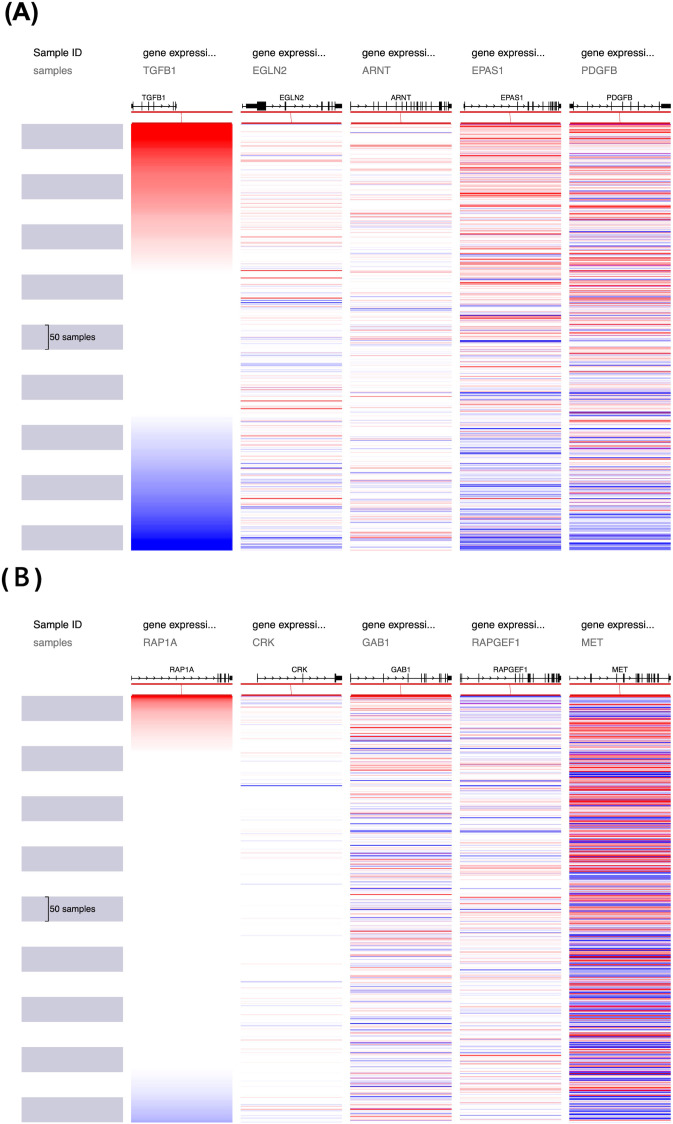
(A). In the KIRC cohort, GRMs display consistent block-like red–blue patterns across metastatic samples, indicating strong module-level co-expression among the five constituent genes. Top-ranked GRMs across five genes (ten pairwise combinations) show mean absolute PCC and SRC values of 0.185 and 0.171, respectively. **(B)**. In the KIRC cohort, the two lowest-ranked GRMs display heterogeneous and uncoordinated expression patterns, lacking clear gene-level coordination co-expression among the five constituent genes. Bottom-ranked GRMs across five genes (ten pairwise combinations) show mean absolute PCC and SRC values of 0.027 and 0.050, respectively.

The two highest-ranked GRMs were summarized using five representative genes, and their expression patterns were visualized with the UCSC Xena browser. These top-ranked GRMs display consistent multi-gene co-variation across metastatic KIRC samples (Fig 4A), whereas the two lowest-ranked GRMs exhibit irregular and uncoordinated expression (Fig 4B).

We quantified gene expression concordance by comparing the top‑ and bottom‑ranked GRMs across five genes, yielding ten pairwise combinations. Concordance was evaluated using Pearson (PCC) and Spearman rank (SRC) correlation coefficients. The two highest‑ranked GRMs showed greater agreement, with mean absolute PCC and SRC values of 0.185 and 0.171, respectively. In contrast, the two lowest‑ranked GRMs demonstrated markedly weaker associations, with mean absolute PCC and SRC values of 0.027 and 0.050.

These qualitative differences indicate that top ranked GRMs capture collaboratively regulated gene programs active in metastasis, whereas bottom ranked GRMs lack association with metastatic expression profiles.

### Generalizability of GRM Identification across Cancer Types

To evaluate the generalizability of the proposed framework, we applied the same GRM ranking and visualization pipeline using the UCSC Xena browser to an independent metastatic lung adenocarcinoma (LUAD) cohort from MET500.

**Fig 5:**
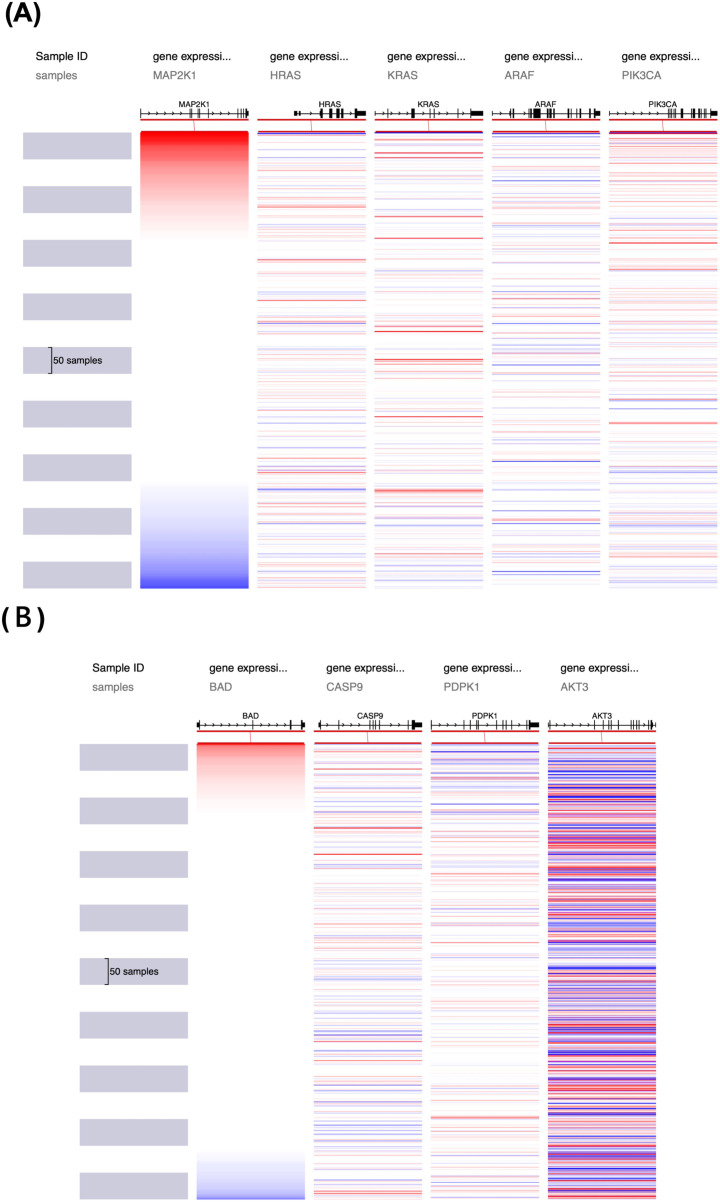
(A). In the LUAD cohort, GRMs display consistent block-like red–blue patterns across metastatic samples, indicating strong module-level co-expression among the five constituent genes. Top-ranked GRMs across five genes (ten pairwise combinations) show mean Pearson and Spearman correlation coefficients of 0.175 and 0.173, respectively. **(B)**. In the LUAD cohort, *the lowest-ranked* GRMs display heterogeneous and uncoordinated expression patterns, lacking clear gene-level coordination co-expression among the five constituent genes. The second lowest‑ranked GRM comprises a *distinct gene set* and is therefore not included in the heatmap. Bottom-ranked GRMs across four genes (six pairwise combinations) show mean Pearson and Spearman correlation coefficients of 0.027 and 0.050, respectively.

The two highest-ranked GRMs show consistent multi‑gene co‑variation across metastatic LUAD samples (Fig 5A), in contrast to the irregular and uncoordinated expression observed among bottom‑ranked GRMs (Fig 5B).

Correlation was assessed using the PCC and SRC. The results showed that top-ranked GRMs exhibited higher concordance, with mean PCC and SRC values of 0.175 and 0.173, respectively. In contrast, the lowest-ranked GRMs displayed markedly weaker associations, with mean PCC and SRC values of 0.054 and 0.050, respectively. These results highlight a clear distinction in expression coherence between high- and low-ranking GRMs.

This clear separation mirrors the observations in KIRC and confirms that the method consistently identifies coordinated regulatory modules across distinct cancer types. These results support our claim that the proposed approach constitutes a general framework applicable beyond a single tumor context.

### Assessment of Cooperative Effects among Top‑ and Bottom‑Ranked GRMs

We performed multivariable Cox proportional hazards regression adjusted for key clinical covariates, including age, sex, and TNM stage. The analysis evaluated cooperative effects among four gene combinations derived from the top and bottom ranked GRMs (Supplementary File 7).

The top‑ranked GRMs exhibited a HR of approximately 2.11, with Benjamini–Hochberg (BH)–adjusted p‑values on the order of <10 ⁻ ⁵. In contrast, the bottom‑ranked GRMs showed an HR of approximately 1.49, with BH‑adjusted p‑values on the order of 2.2 × 10 ⁻ ⁴, indicating a substantially stronger relative risk associated with the top‑ranked GRMs. These results suggest that top‑ranked GRMs confer a higher metastasis‑associated risk than lower‑ranked GRMs after adjustment for clinical covariates, including gender, age and TNM staging.

Multivariable Cox regression was performed across three independent KIRC cohorts (Table 4), evaluating three predefined gene groups: (i) *COL1A1, DCN, FBLN1*, and *POSTN;* (ii) *ABCG1, HAVCR2, CD14*, and *TGFA;* and (iii) *VHL, PBRM1, BAP1*, and *SETD2*. Gene sets (i) and (ii) showed statistically significant associations (BH p-value < 0.05) with outcome, although their hazard ratios were close to 1, indicating small effect sizes (Supplementary File 8). In contrast, gene set (iii) did not show a significant association. These findings further support the effectiveness of our approach in identifying metastasis‑associated GRMs.

## Conclusion

We have tested our hypothesis, which suggests that the 4-node GRMs consisting of genetic elements are key candidates for metastasis-associated gene modules. By developing an integrated approach that combines the subgraph method and CFA, we uncovered metastasis-associated GRMs. Validation through enrichment analysis, drug–target gene insights, survival data, and comparison with previously published work demonstrates its potential for identifying metastasis-associated target genes and discovering therapeutic drug candidates.

### Limitation and future works

Despite the promising results, some limitations must be acknowledged. One key limitation is that our approach does not consider the rank combination method due to the constraints imposed by the cancer driver genes (*cdg*) scoring method, which allows only a limited number of ranks. To address this issue, we suggest incorporating additional genetic categories, such as passenger driver genes and essential genes, into the scoring system to refine the evaluation. Additionally, expanding the number of metastasis databases beyond three could help mitigate the issue of having too many tiers.

Furthermore, incorporating external datasets, such as Firebrowse (http://firebrowse.org/), could further improve the ranking accuracy. Firebrowse offers access to comprehensive TCGA data, including detailed cancer staging information, which could enrich our analyses and provide more robust results.

Future research could also explore the scalability issue in constructing larger GRMs. Specifically, we note that multiple 4 node GRMs can, in principle, be merged to form 5 node or 6 node GRMs—referred to as Coupled Motif Structures (CMS) [64]—which may help address scalability challenges and facilitate the identification of larger metastasis associated regulatory modules. For example, one type of CMS considered involves merging the top ranked GRMs. Once constructed, these CMSs provide a means to reconstruct the global architecture of the larger regulatory network through a bottom up approach. However, we also point out that expanding GRM size introduces substantial missing data issues as additional genetic elements are incorporated, which in turn limits the practical scalability of the present approach.

To experimentally assess whether the predicted GRMs function as coherent regulatory modules rather than collections of independent genes. Specifically, we selected the top two GRMs for focused functional validation. For each GRM, which is composed of four genes, we propose combinatorial perturbation experiments using multiplex siRNA or CRISPRi approaches to simultaneously suppress multiple GRM members. Functional readouts including cell migration, invasion, and EMT‑related markers will be compared across single‑gene, partial, and full GRM perturbations. A non‑additive or synergistic phenotypic effect observed under full GRM perturbation, exceeding the summed effects of individual gene perturbations, would indicate functional cooperativity at the module level.

Finally, the findings suggest that the developed method could be applied to predict the relevance of new GRMs inferred from the KIRC cancer cohort. By calculating ranks using the four genetic component scores—*FC, HR, cdg*, and *Occ*—GRMs can be ranked and assessed for their potential association with tumor metastasis, providing valuable insights for future cancer research and drug development.

Our comprehensive analysis demonstrates that the method developed for ranking and evaluating GRMs in the context of KIRC is effective in isolating tumor metastasis-associated GRMs. By integrating multiple scoring methods and using GOEA, hallmark annotations, drug-target information, and survival data, we provide a robust framework for identifying significant GRMs related to cancer formation and metastasis.

## Supporting information

S1 File The set of 4-node GRMs identified in the renal kidney cancer network. (XLSX)

S2 File The complete set of all 199 possible 4-node gene regulatory modules. (PDF)

S3 File The results of enrichment analysis for the highest two ranked 4-node GRMs across all 11 combinations of the four scoring methods. (XLSX)

S4 File The results of enrichment analysis for the lowest two ranked 4-node GRMs across all 11 combinations of the four scoring methods. (XLSX)

S5 File Results of the Jaccard index (*JI*) between the weighted and average score approaches, comparing (i) the top five and bottom five GRMs, and (ii) the top ten and bottom ten GRMs, across all 11 combinations of the four scoring methods. (DOCX)

S6 File The results of enrichment analysis for the highest two and lowest two ranked 3-node GRMs using four scoring methods. (XLSX)

S7 File The results from multivariable Cox proportional hazards regression analyses of the KIRC cohorts, adjusted for key clinical covariates including age, gender, and TNM stage. (XLSX)

S8 File Multivariable Cox proportional hazards regression results from three independent KIRC cohorts, adjusted for age, gender, and TNM stage. (CSV)

S9 File The adjacency matrix for the KIRC network. (TXT)
