# Supplementary material for: Identifying gene regulation modules associated with tumor metastasis using a network decomposition approach and combinatorial fusion analysis
Source: PLoS One. 2026 Jun 26;21(6):e0337873. doi: 10.1371/journal.pone.0337873 (PMC13308872; doi:10.1371/journal.pone.0337873)
Supplement: S2 File — (PDF) [file pone.0337873.s002.pdf]

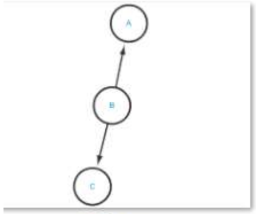

3_6.png

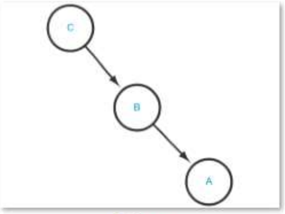

3_12.png

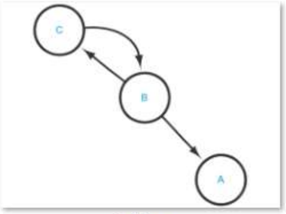

3_14.png

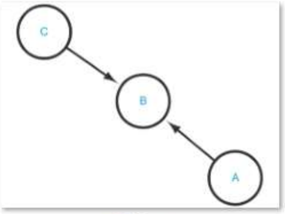

3_36.png

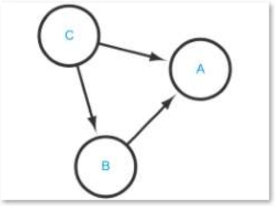

3_38.png

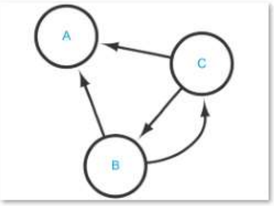

3_46.png

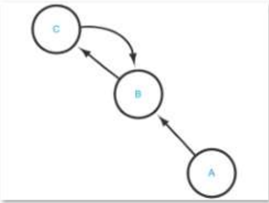

3_74.png

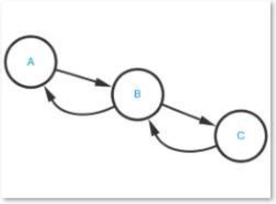

3_78.png

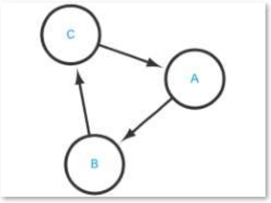

3_98.png

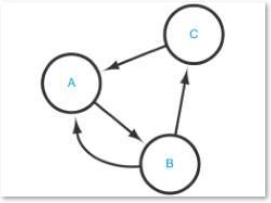

3_102.png

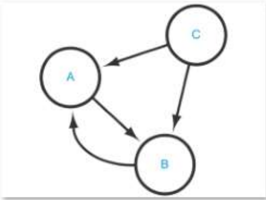

3_108.png

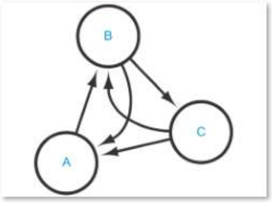

3_110.png

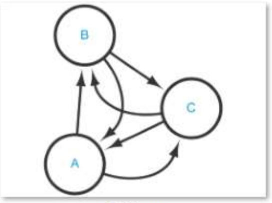

3_238.png

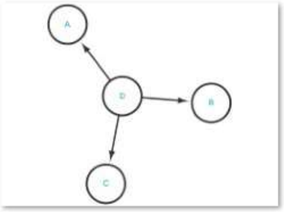

4_14.png

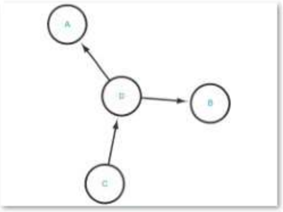

4_28.png

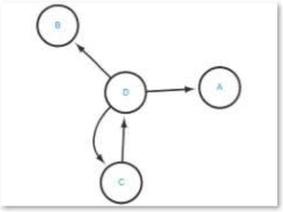

4_30.png

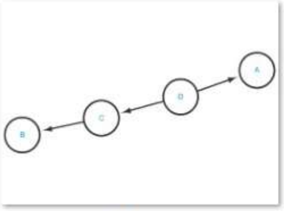

4_74.png

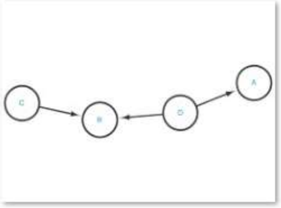

4_76.png

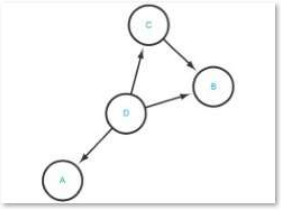

4_78.png

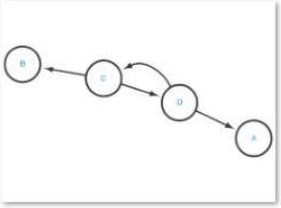

4_90.png

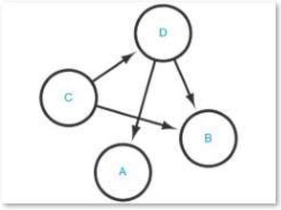

4_92.png

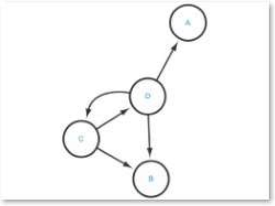

4_94.png

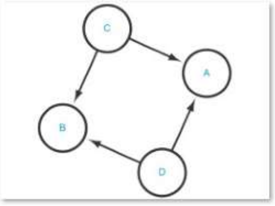

4_204.png

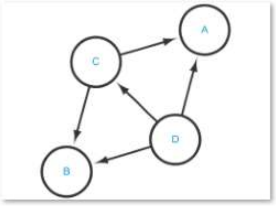

4_206.png

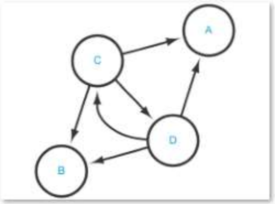

4_222.png

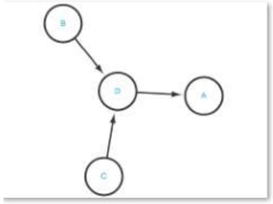
4_280.png

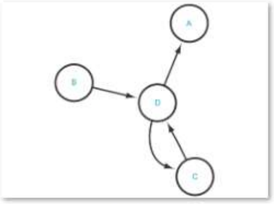
4_282.png

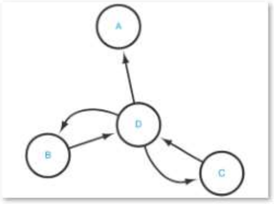

4_286.png

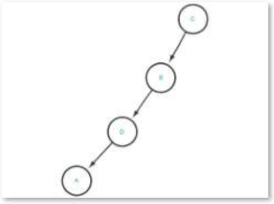
4_328.png

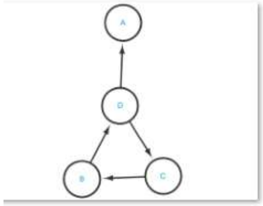
4_330.png

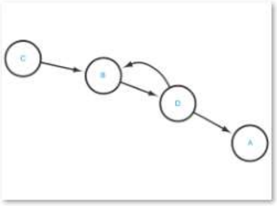
4_332.png

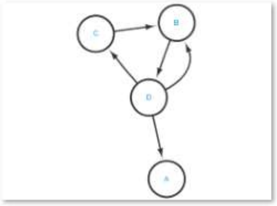
4_334.png

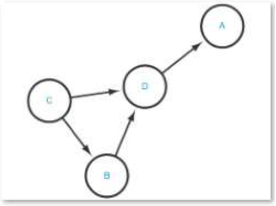

4_344.png

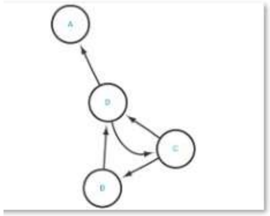
4_346.png

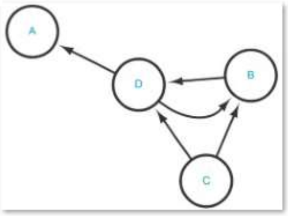

4_348.png

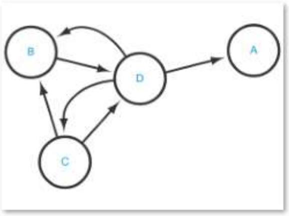

4_350.png

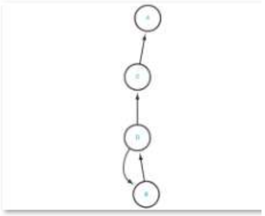
4_390.png

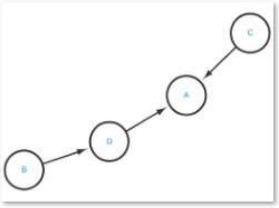
4_392.png

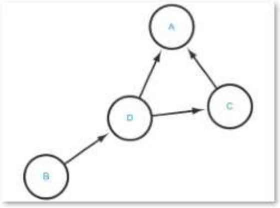
4_394.png

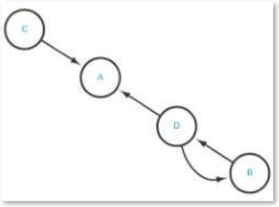
4_396.png

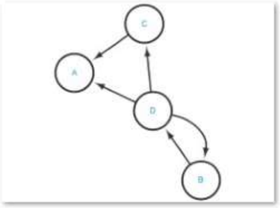
4_398.png

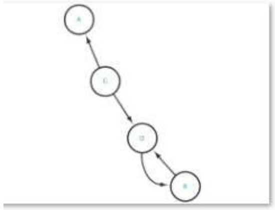
4_404.png

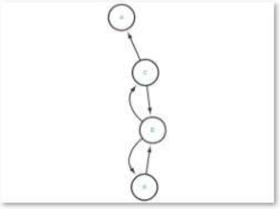
4_406.png

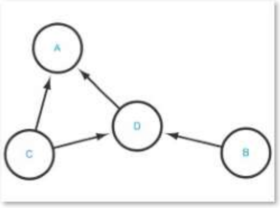

4_408.png

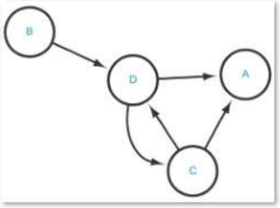

4_410.png

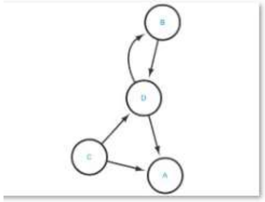

4_412.png

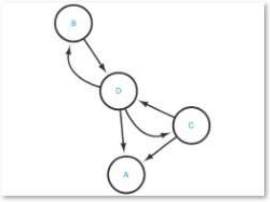

4_414.png

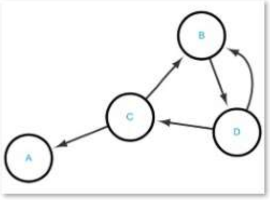

4_454.png

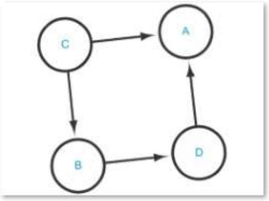

4_456.png

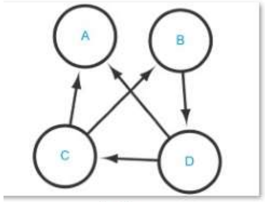

4_458.png

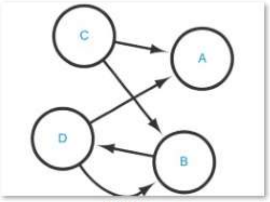

4_460.png

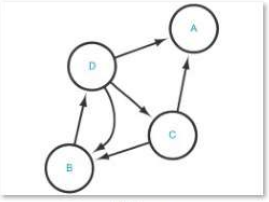

4_462.png

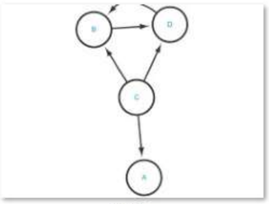

4_468.png

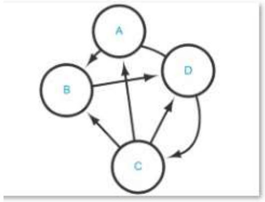

4_470.png

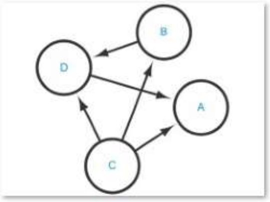

4_472.png

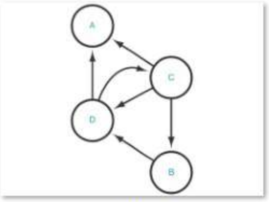

4_474.png

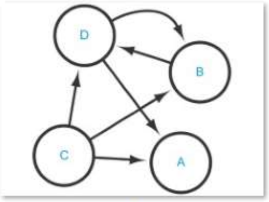

4_476.png

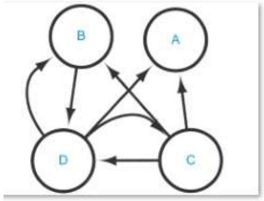

4_478.png

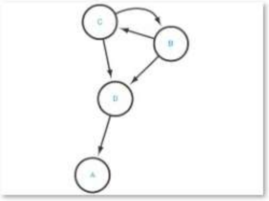

4_856.png

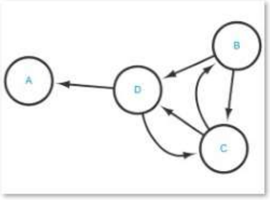

4_858.png

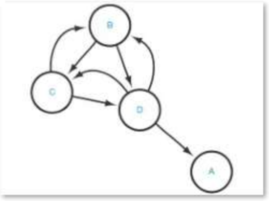

4_862.png

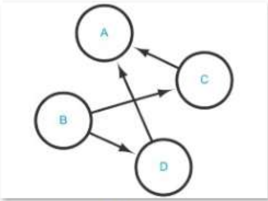

4_904.png

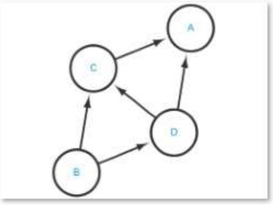

4_906.png

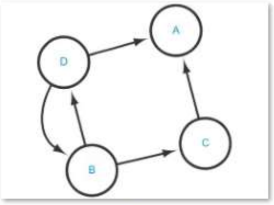

4_908.png

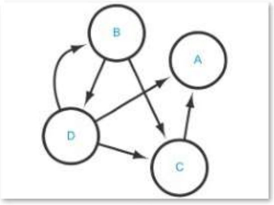

4_910.png

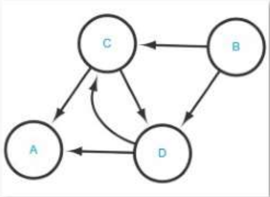

4_922.png

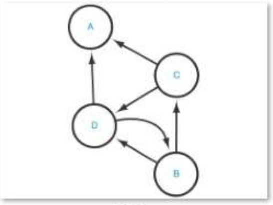

4_924.png

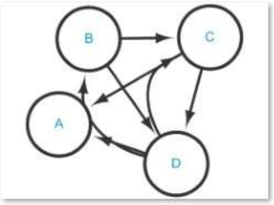

4_926.png

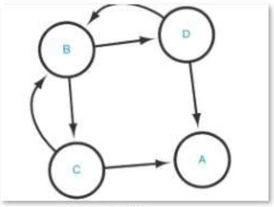

4_972.png

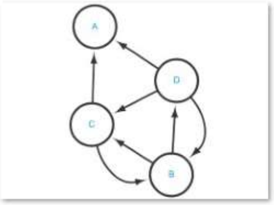

4_974.png

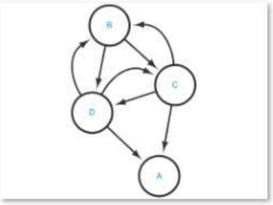

4_990.png

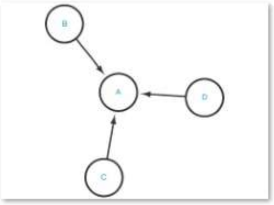

4_2184.png

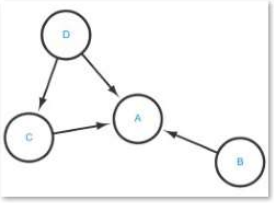

4_2186.png

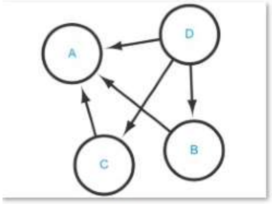

4_2190.png

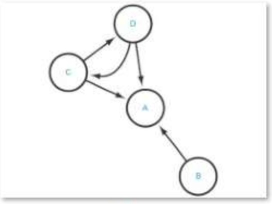

4_2202.png

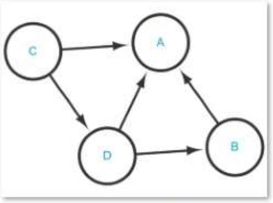

4_2204.png

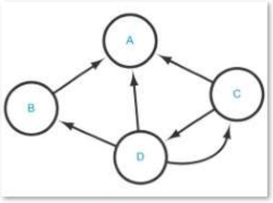

4_2206.png

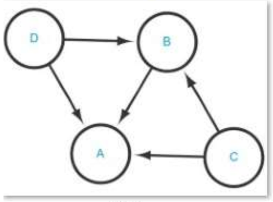

4_2252.png

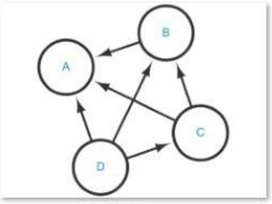

4_2254.png

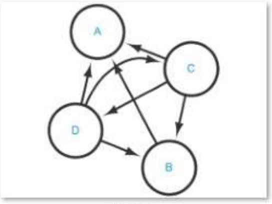

4_2270.png

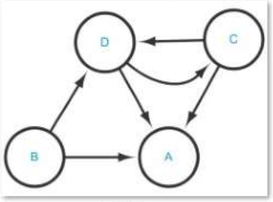

4_2458.png

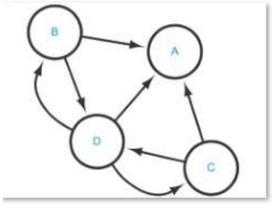

4_2462.png

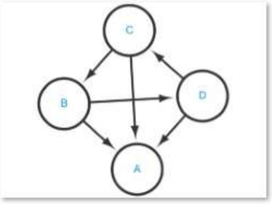

4_2506.png

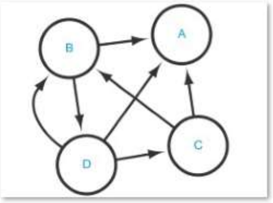

4_2510.png

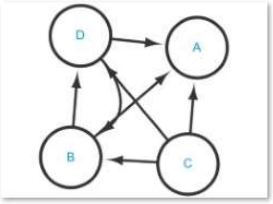

4_2524.png

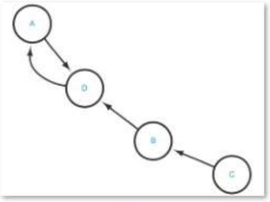

4_4424.png

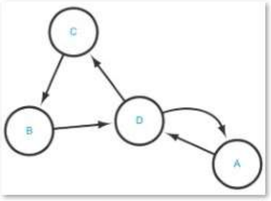

4_4426.png

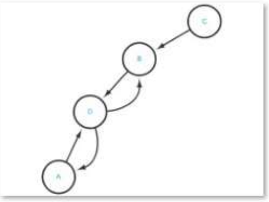

4_4428.png

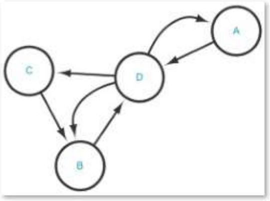

4_4430.png

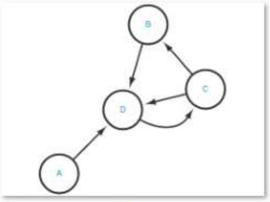

4_4434.png

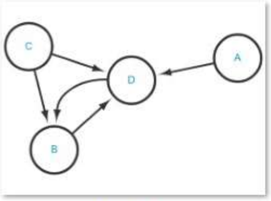

4_4436.png

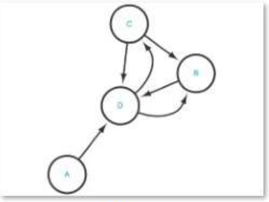

4_4438.png

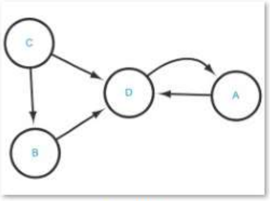

4_4440.png

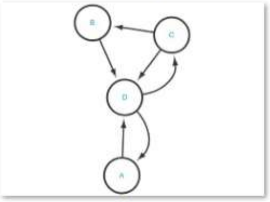

4_4442.png

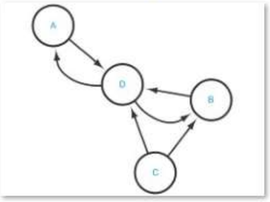

4_4444.png

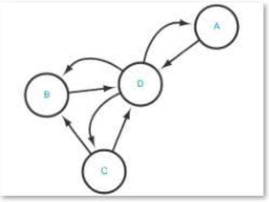

4_4446.png

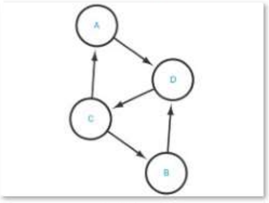

4_4546.png

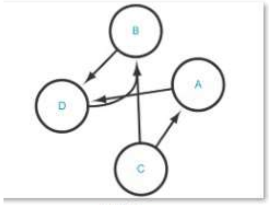

4_4548.png

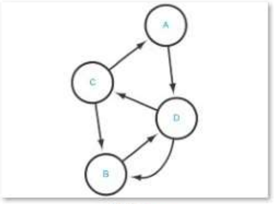

4_4550.png

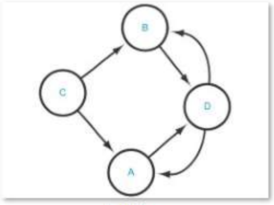

4_4556.png

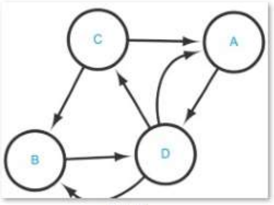

4_4558.png

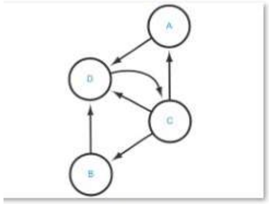

4_4562.png

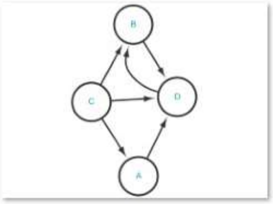

4_4564.png

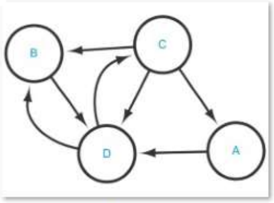

4_4566.png

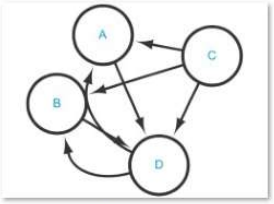

4_4572.png

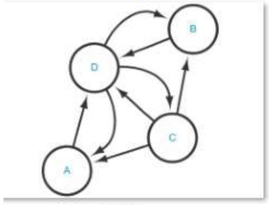

4_4574.png

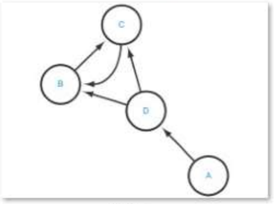

4_4678.png

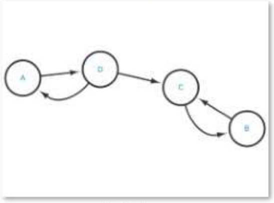

4_4682.png

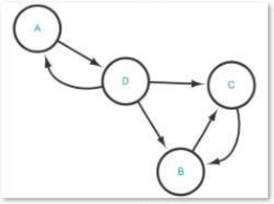

4_4686.png

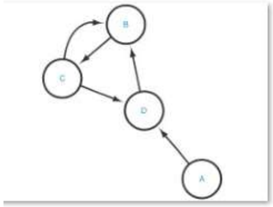

4_4692.png

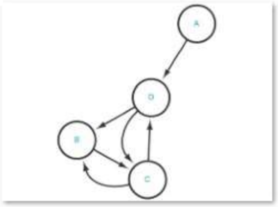

4_4694.png

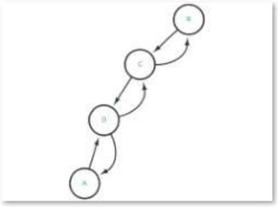

4_4698.png

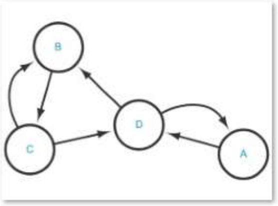

4_4700.png

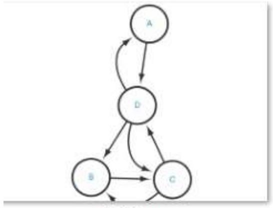

4_4702.png

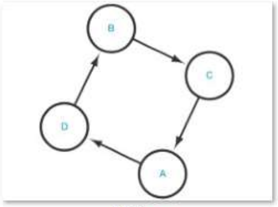

4_4740.png

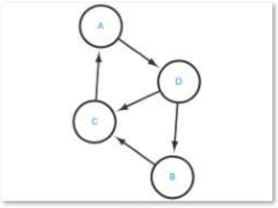

4_4742.png

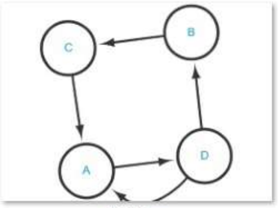

4_4748.png

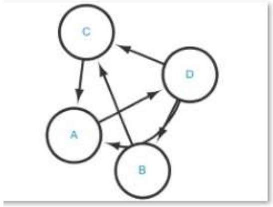

4_4750.png

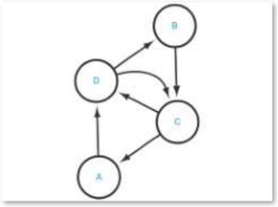

4_4758.png

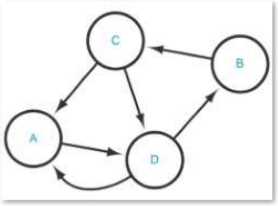

4_4764.png

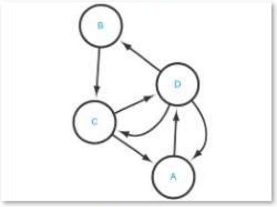

4_4766.png

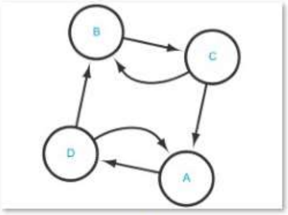

4_4812.png

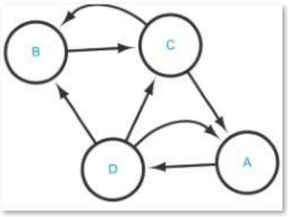

4_4814.png

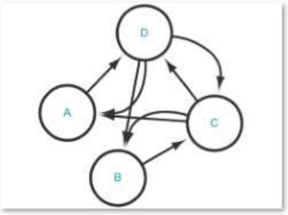

4_4830.png

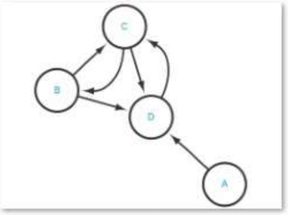

4_4946.png

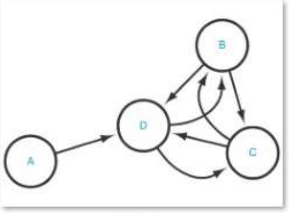

4_4950.png

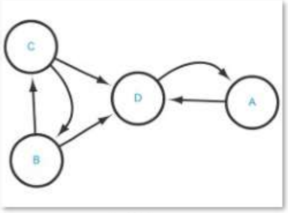

4_4952.png

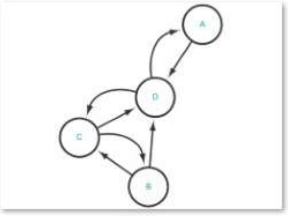

4_4954.png

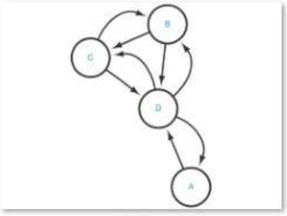

4_4958.png

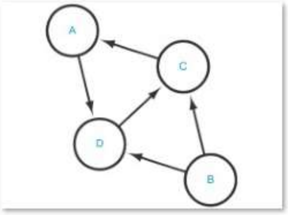

4_4994.png

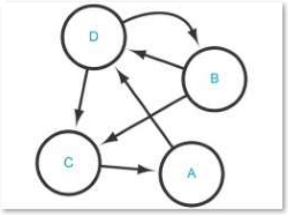

4_4998.png

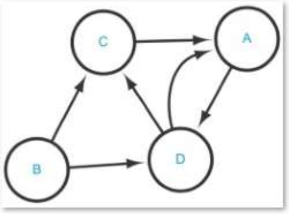

4_5002.png

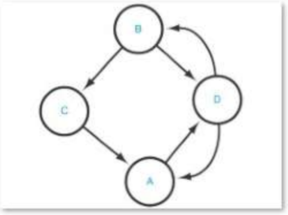

4_5004.png

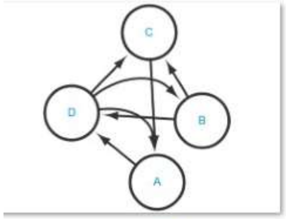

4_5006.png

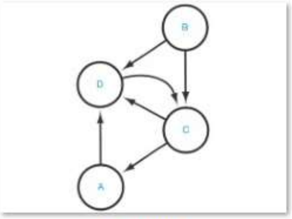

4_5010.png

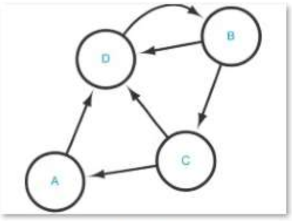

4_5012.png

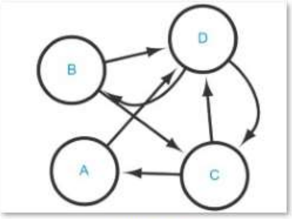

4_5014.png

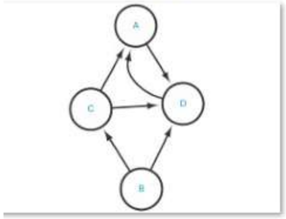

4_5016.png

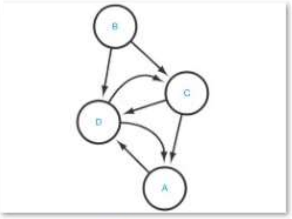

4_5018.png

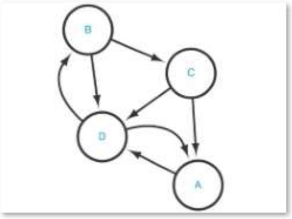

4_5020.png

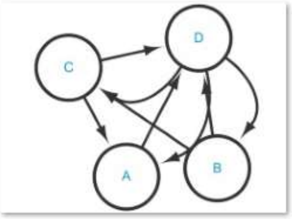

4_5022.png

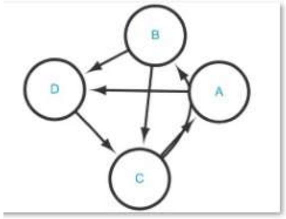

4_5058.png

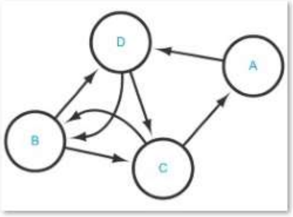

4_5062.png

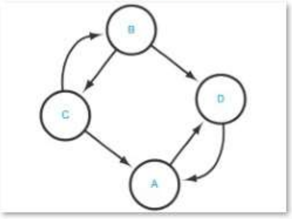

4_5064.png

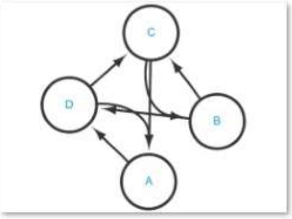

4_5066.png

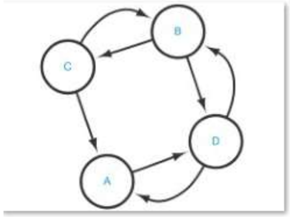

4_5068.png

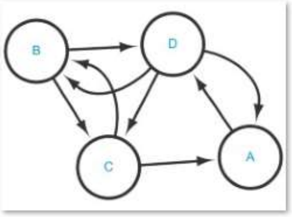

4_5070.png

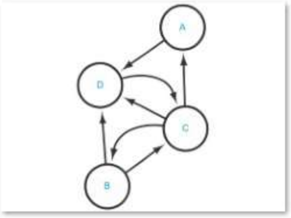

4_5074.png

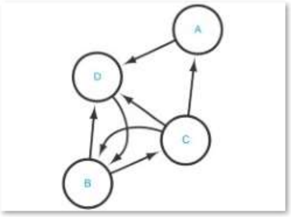

4_5076.png

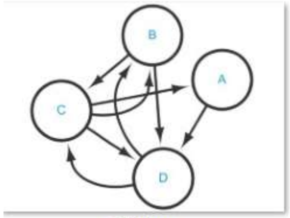

4_5078.png

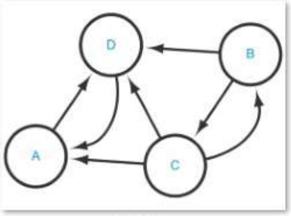

4_5080.png

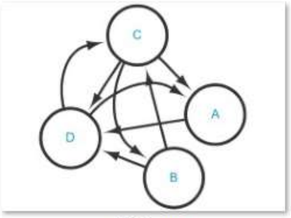

4_5082.png

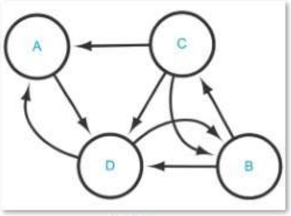

4_5084.png

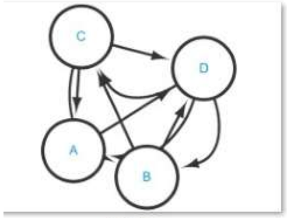

4_5086.png

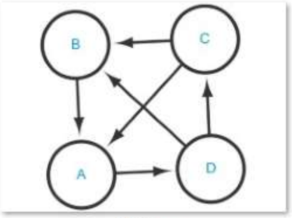

4_6342.png

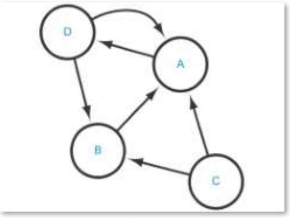

4_6348.png

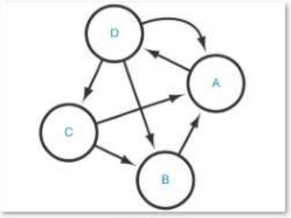

4_6350.png

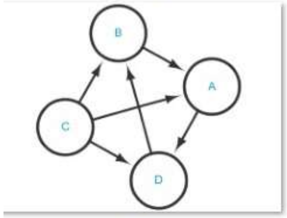

4_6356.png

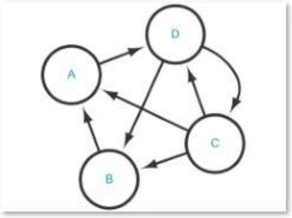

4_6358.png

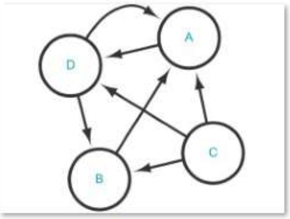

4_6364.png

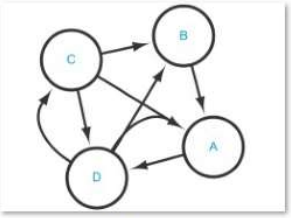

4_6366.png

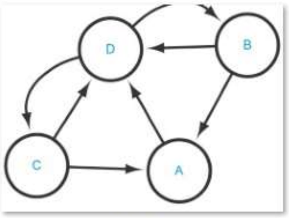

4_6550.png

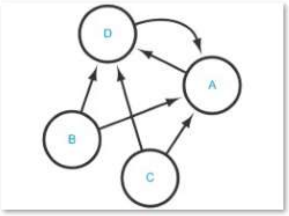

4_6552.png

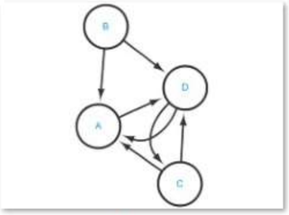

4_6554.png

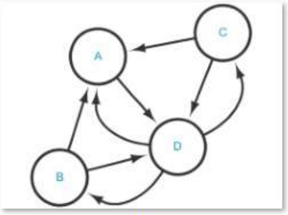

4_6558.png

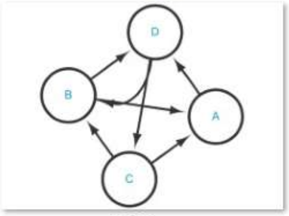

4_6598.png

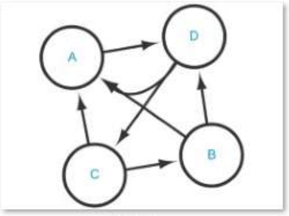

4_6602.png

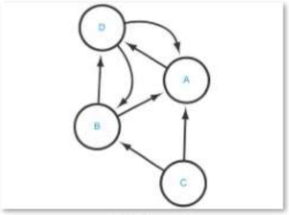

4_6604.png

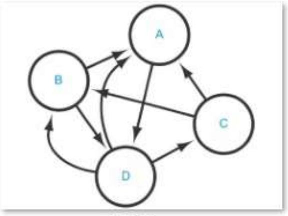

4_6606.png

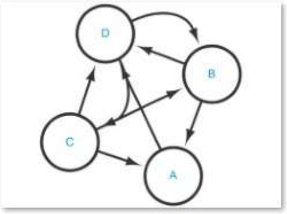

4_6614.png

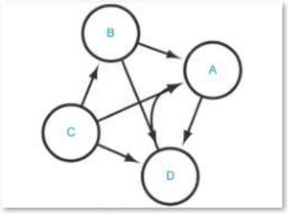

4_6616.png

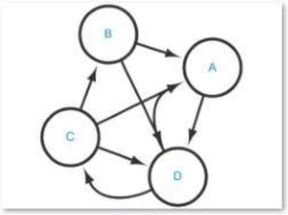

4_6618.png

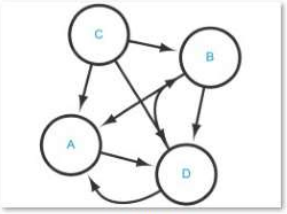

4_6620.png

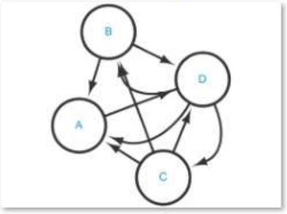

4_6622.png

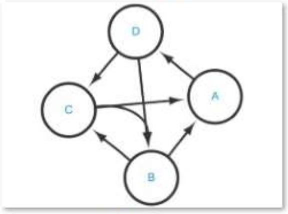

4_6854.png

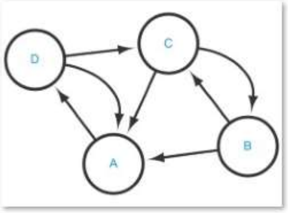

4_6858.png

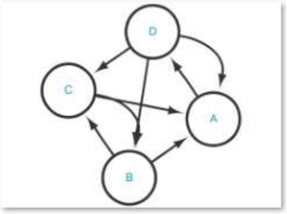

4_6862.png

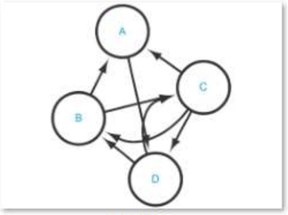

4_6870.png

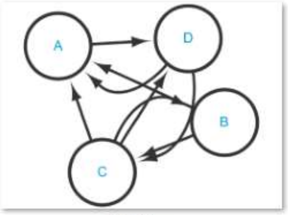

4_6874.png

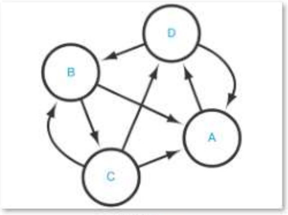

4_6876.png

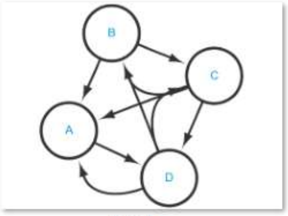

4_6878.png

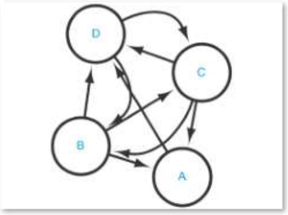

4_7126.png

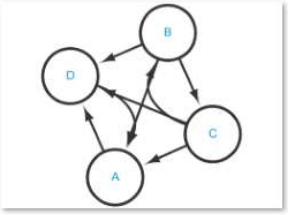

4_7128.png

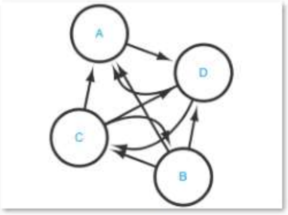

4_7130.png

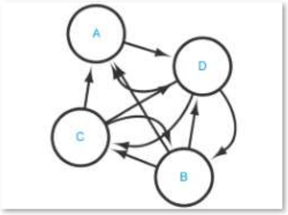

4_7134.png

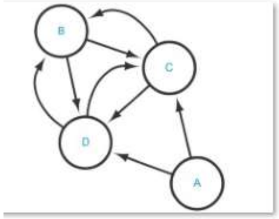

4_13142.png

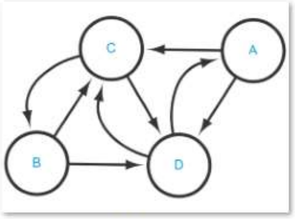

4_13146.png

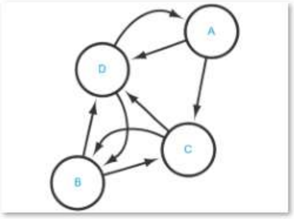

4_13148.png

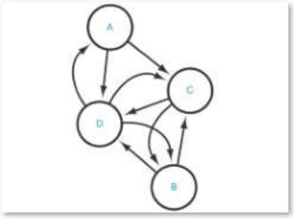

4_13150.png

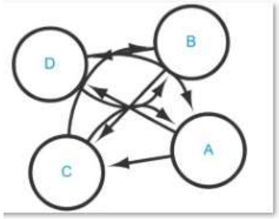

4_13260.png

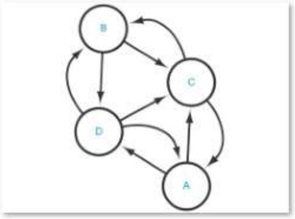

4_13262.png

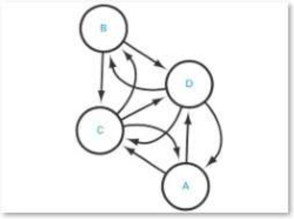

4_13278.png

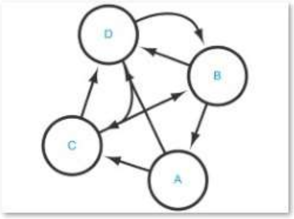

4_14678.png

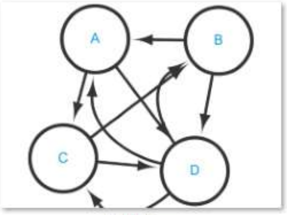

4_14686.png

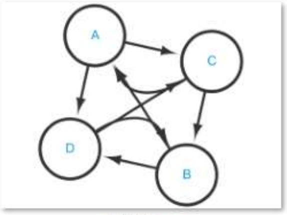

4_14790.png

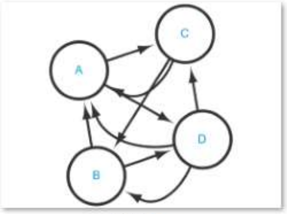

4_14798.png

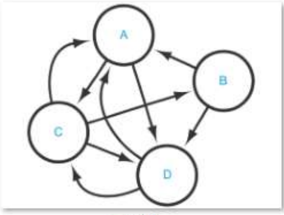

4_14810.png

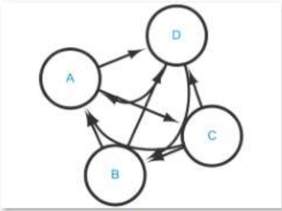

4_14812.png

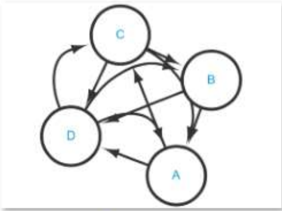

4_14814.png

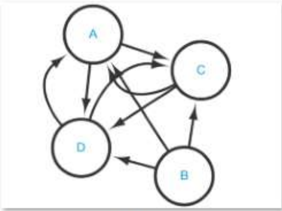

4_15258.png

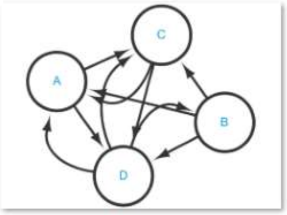

4_15262.png

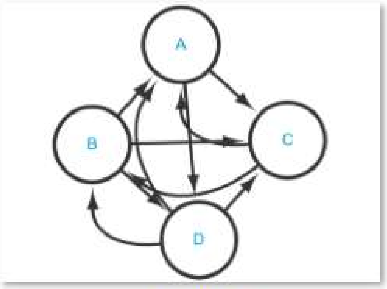

4_15310.png

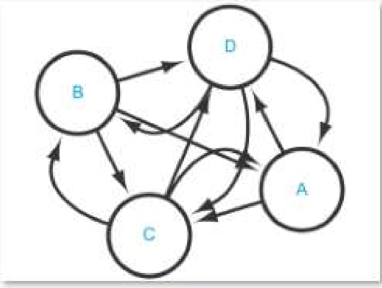

4_15326.png

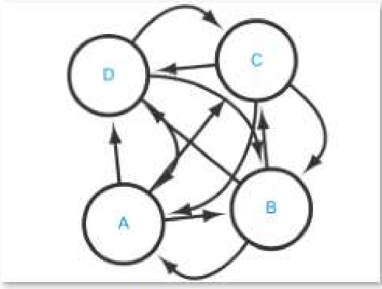

4_31710.png
