# Supplementary material for: Identifying gene regulation modules associated with tumor metastasis using a network decomposition approach and combinatorial fusion analysis
Source: PLoS One. 2026 Jun 26;21(6):e0337873. doi: 10.1371/journal.pone.0337873 (PMC13308872; doi:10.1371/journal.pone.0337873)
Supplement: S5 File — (DOCX) [file pone.0337873.s005.docx]

Supplementary File 5. Results of the Jaccard index (*JI*) between the weighted and average score approaches, comparing (i) the top five and bottom five GRMs, and (ii) the top ten and bottom ten GRMs, across all 11 combinations of the four scoring methods.

| Score combination | *JI*, top 5 GRMs | *JI,* bottom 5 GRMs | *JI,* top 10 GRMs | *JI,* top 10 GRMs |
| --- | --- | --- | --- | --- |
| *FC_HR_occ_cdg* | 0.667 | 0.667 | 0.667 | 0.818 |
| *FC_HR_occ* | 1 | 1 | 0.667 | 0.667 |
| *FC_HR_cdg* | 0.667 | 1 | 1 | 0.667 |
| *FC_occ_cdg* | 0.667 | 0.667 | 0.818 | 0.538 |
| *HR_occ_cdg* | 0.667 | 0.429 | 0.667 | 0.818 |
| *FC_HR* | 1 | 1 | 1 | 1 |
| *FC_occ* | 1 | 1 | 1 | 1 |
| *FC_cdg* | 0.667 | 0.429 | 0.667 | 0.818 |
| *HR_occ* | 1 | 1 | 1 | 1 |
| *HR_cdg* | 1 | 1 | 0.818 | 1 |
| *occ_cdg* | 1 | 1 | 1 | 1 |
